# The COVID-19 thrombus: distinguishing pathological, mechanistic, and phenotypic features and management

**DOI:** 10.1007/s11239-024-03028-4

**Published:** 2024-08-23

**Authors:** Richard C. Becker, Udaya S. Tantry, Muhammad Khan, Paul A. Gurbel

**Affiliations:** 1 Cardiovascular Center, University of Cincinnati College of Medicine, 231 Albert Sabin Way, Cincinnati, OH 45267 USA; 2 Sinai Center for Thrombosis Research and Drug Development, Baltimore, USA; 3 Division of General Internal Medicine, University of Cincinnati College of Medicine, Cincinnati, USA

**Keywords:** COVID-19-associated thrombosis, Microvascular thrombosis, Macrovascular thrombosis

## Abstract

A heightened risk for thrombosis is a hallmark of COVID-19. Expansive clinical experience and medical literature have characterized small (*micro*) and large *(macro*) vessel involvement of the venous and arterial circulatory systems. Most events occur in patients with serious or critical illness in the *hyperacute* (first 1–2 weeks) or *acute phases* (2–4 weeks) of SARS-CoV-2 infection. However, thrombosis involving the venous, arterial, and microcirculatory systems has been reported in the *subacute* (4–8 weeks), *convalescent* (> 8–12 weeks) and *chronic phases* (> 12 weeks) among patients with mild-to-moderate illness. The purpose of the current focused review is to highlight the distinguishing clinical features, pathological components, and potential mechanisms of venous, arterial, and microvascular thrombosis in patients with COVID-19. The overarching objective is to better understand the proclivity for thrombosis, laying a solid foundation for screening and surveillance modalities, preventive strategies, and optimal patient management.

**Graphical abstract:**

Thrombosis involving the arterial, venous, and microvascular circulatory systems is a hallmark of COVID-19 contributing to organ injury, morbidity, and mortality. The COVID-19 thrombus has unique and distinct characteristics. Among them are a highly inflammatory signature on a foundation of endothelial cell inflammation and neutrophil extracellular traps (NETs). While the SARS-CoV-2 virus and resulting host immunoinflammatory response to this pathogen provide an underpinning for vascular events, the virus spike protein is necessary and likely sufficient for acute, subacute, and potentially in a latent form harbored in adipocytes, vascular endothelial cells, and circulating monocytes potentially contributing to post-infectious atherothrombotic phenotypes.

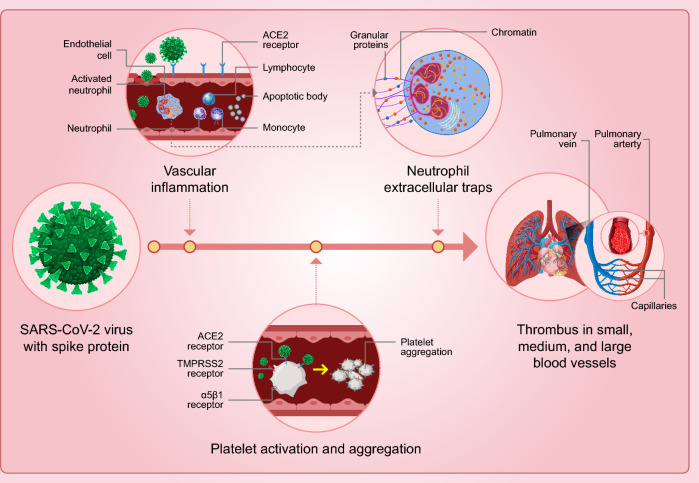

## Highlights

- COVID-19, caused by the SARS-CoV-2 virus is an acquired thrombophila with a high incidence of thrombosis.
- Thrombosis is caused by endothelial cell inflammation and an immuno-inflammatory state.
- Thrombosis occurs in the venous, arterial, and microcirculatory systems and can involve most organs and organ systems.
- The long-term sequela of COVID-19 could include a heightened risk for atherosclerosis and cardiovascular events.
- There is a need for indepth investigation of the natural history of COVID-19 to include long-COVID also refered to as post-COVID conditions.

## Introduction

COVID-19 predisposes to thrombotic and thromboembolic events, owing to excessive inflammation, endothelial cell activation and injury, platelet activation, coagulation protease activation, impaired fibrinolysis, culminating in both a local and systemic thrombophilic state (reviewed in Gorog) [1]. A complex and highly dynamic infectious, immune, autoimmune, neurological, and hemodynamic environment characterizes the *hyperacute* (first 1–2 weeks), *acute* (2–4 weeks)*, subacute* (4–8 weeks)*, convalescent* (˃8–12 weeks), and chronic (˃12 weeks) phases of infection with attendant risk for thrombosis involving the venous, arterial, and microvascular circulatory systems (reviewed in Becker RC) [2].

**Fig. 1:**
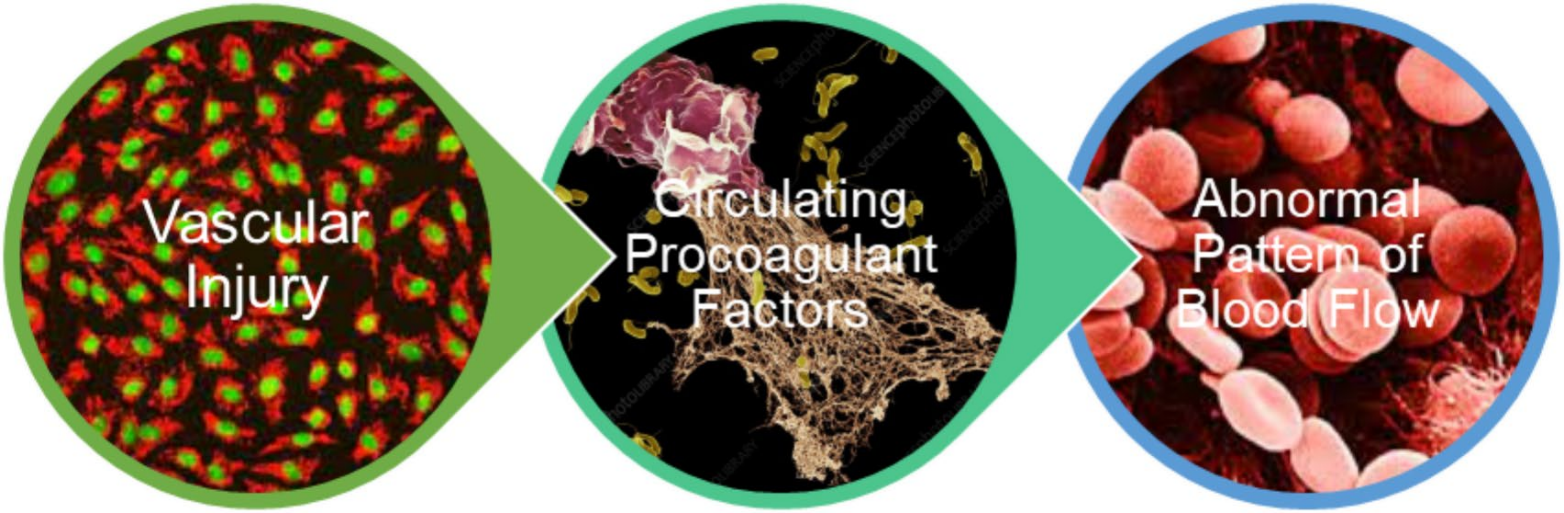
Virchow’s triad and dyad: the traditional Virchow’s triad includes abnormalities in the vasculature, blood, and flow as determinants of thrombosis. In some settings like COVID-19 the extent of vascular injury and inflammation, coupled with highly prothrombotic factors within the circulating blood through small, medium, and large-size vessels may be sufficient to cause thrombosis

Applying Virchow’s triad to COVID-19 provides several clues to its proclivity for thrombosis- vascular injury, upregulation of platelet activation and coagulation, and impaired endothelial cell facilitated vascular repair, surface anticoagulant properties, vascular-responsiveness, and fibrinolytic capacity. In addition to direct virus-associated events, autoimmune responses are believed to play a large role in COVID-19 thrombosis and thromboembolism. The extent of vascular inflammation, injury, and dysfunction, coupled with circulating procoagulant factors creates an environment or *Virchow’s dyad* (COVID Dyad) that may be enough to explain the frequency of events-both subclinical and clinical [2] (Fig. 1).

Because thrombosis is more common in COVID-19 than diseases caused by other coronaviruses and influenza A [3–5], we sought to summarize the pathophysiology of thrombosis, thrombus architecture, and contribution of the immune-inflammatory tissue and systemic environments to widely observed phenotypes. We also describe the temporal relationship between the acute infection and thrombosis, potential mechanisms of long-COVID and thrombosis, provide an overview of prevention-based antithrombotic therapy trials, and propose a pragmatic surveillance model for patients at risk for future atherothrombotic events.

## The COVID-19 virus

The trimeric SARS-CoV-2 surface spike (S) protein consists of three S1S2 heterodimers that bind the cellular receptor angiotensin converting enzyme (ACE) 2 and mediate fusion at the viral and host cellular membranes through a pre-to-post fusion conformational change. For ACE2-mediated cell entry of SARS-CoV-2, co-expression of one more protein—transmembrane protease serine 2 (TMPRSS2), is essential [6]. The S1 subunit contains the receptor-binding domain (RBD). After receptor binding, proteolytic processing causes a conformational change in S1. The available information obtained by cryo-electron microscopy shows that only one receptor-binding domain binds ACE2 and adopts an upward conformation. Moreover, binding to the receptors opens the receptor binding domain of S1 and promotes the release of the S1-ACE2 complex and S1 monomers (Reviewed in Becker RC) [7].

The initial step in SARS-CoV-2 infection, binding to the ACE2 receptor, is both unique and distinct to this group of viral pathogens [8]. Because ACE2 receptors are present in all vascular endothelial cells regardless of host tissue, organ, or organ system, a potential nidus for vascular injury is common and widespread among persons with COVID-19 [9]. The extent of injury correlates with the burden of virus, density of binding sites, and an immunoinflammatory response that is both host and tissue-specific [10]. While spike protein containing the S1 and S2 regions can bind to endothelial cells, the receptor binding domain (RBD) found within the S1 subunit requires the presence of a high density of ACE2 receptors for an interaction [11].

## The initial immuno-inflammatory response to infection

**Fig. 2:**
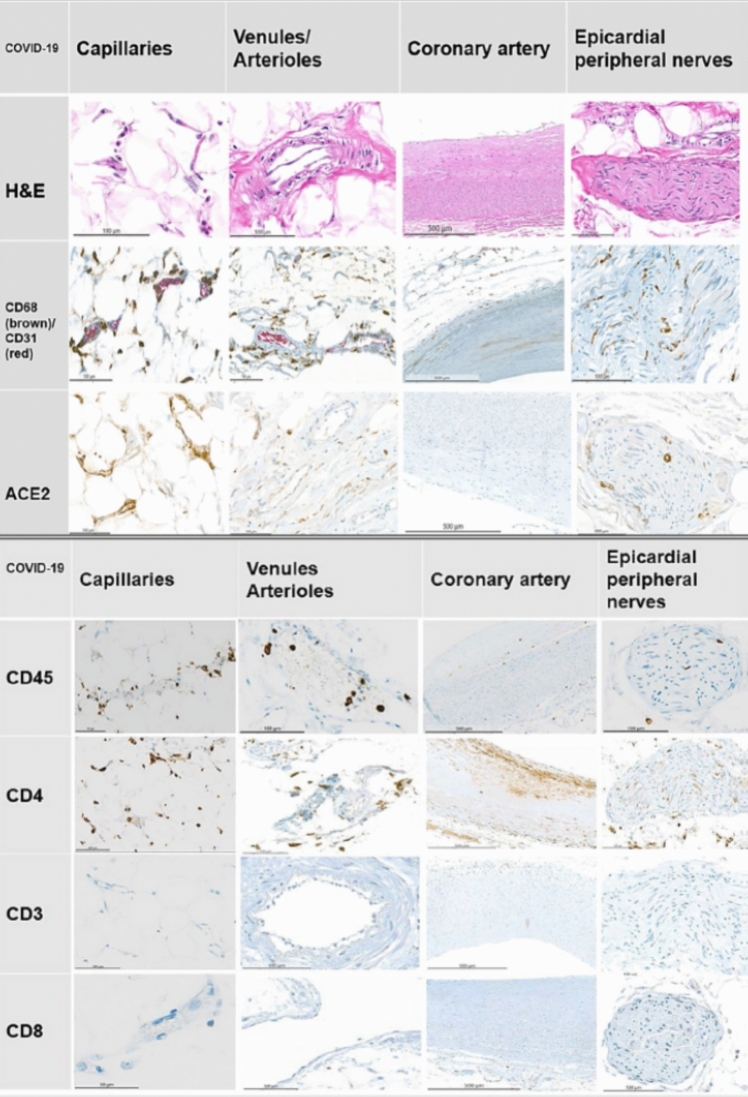
Endotheliitis and neuritis: representative immunostains and H&E sections from COVID-19-positive patients. An endotheliitis of small vessels and a neuritis with prevalence of CD4-positive and CD68-positive inflammatory cells are demonstrated along with a strong expression of ACE2 receptor. The red (CD31) stains show endothelial cell, the brown reaction products (all other stains) mark positively stained inflammatory cells. Relevant immunostains (CD68, CD45, CD68, ACE2) show a trend being the highest in the capillaries and venules and less pronounced in the main coronary arteries in a semi-quantitative analysis. From Maccio U. EBioMedicine 2021;63: 103,182: with permission

SARS-CoV-2 is an intracellular pathogen. Accordingly, TH1 cells, which promote cytotoxic T cells and cell-mediated immunity are a major source of immune defenses, triggering complement activation, followed by an acute inflammatory response, TH2 cell activation and adaptive antibody production. Thrombosis occurring at the microvascular level is largely triggered by activated neutrophils and release of inflammatory mediators with the primary objective to localize infections. A high viral load coupled with poorly regulated local and systemic hyperinflammatory responses are collectively responsible for more widespread vascular injury that serves as a powerful nidus for thrombosis. While vascular repair should occur with infection control, other important mechanisms likely explain longer term thrombosis risk. These mechanisms involve persistently activated immune memory cells, humoral autoantibodies directed at dermatan sulfate and surface proteins responsible for maintaining vascular integrity, and reservoirs of virus remnants, particularly spike proteins that have either direct prothrombotic potential or antigenic capacity for producing antibodies that target one or more vital endothelial cell or glycocalyx proteins. The inflammatory, immuno-inflammatory, and auto-immune stages of SARS-CoV-2 infection and related host responses provide a functional characterization of COVID-19 as a continuum of disease that is useful for understanding and investigating prevention and treatment (Fig. 2).

## Preclinical models of SARS-CoV-2 infection and thrombosis

Animal models of SARS-COV-2 infection have been employed as a vital component of a multi-tiered investigational architecture (review in Chu) [12]. The following models have shown promise for translating infection, pathology, thrombosis phenotypes and potential prevention or intervention platforms (Table 1).

**Table 1:** Animals models of SARVS-COV-2 infection

Model	Strengths	Limitations	Translation	Other
Golden hamster	• ACE2 similarity • Lung pathology • Virus transmission • Sensory neuron pathology	• Non-lethal • Infection localized • Reversible pathology	• Masks reduced virus transmission • Virus gene variants • Vaccine studies	• Low cost
Mouse	• Sensitized to SARS-COV-2 by hACE2 expression • Experience with SARS-COV • Neurological involvement		• Virus-dose dependent pathology • Lethality • Virus replication • Survival rates associated with virus and pathology	• Low cost • Accessible • Reagent availability • Rich repertoire of models • Translatability for immune responses?
Ferret	• Naturally susceptible to SARS-COV-2 • Immune response	• Limited severity • Moderate cost	• Transmission • Drug Effect • Innate immune response • Severity of disease	
Non-human primates –Rhesus macaques –Cynomolgus macaques –Marmosets –Baboon	• Susceptible to SARS-COV-2 • Used in vaccine development • Used in antiviral drug development	• Mild- to- moderate infection • High costs	• Response to vaccine • Response to remdesivir • Cellular and humoral response	

A mouse model of SARS-CoV-2 infection demonstrated severe lung pathology and neutrophil-mediated immunopathology [13]. Chemokine-activated neutrophils stimulated proinflammatory gene expression and neutrophil release of their granular contents. A Cd177 high cluster of cells was responsible for neutrophil extracellular trap (NET) formation and consumption of arginine with dampened B cell function.

Employing golden hamsters and rhesus macaque models, Aid et al. [14] demonstrated that SARS-COV-2 challenges stimulated myeloid and inflammatory programs as well as signatures of complement and thrombosis activation (coagulation pathways, platelet activation and aggregation). Recombinant, replication incompetent Ad26 vectors expressing SARS-COV-2 spike constructs prevented clinical disease and attenuated complement activation and coagulation activation. The degree of activation correlated with SARS-COV-2 neutralizing antibody titers.

Ferrets can be infected with SARS-CoV-2 and do transmit infection to contact animals. The severity of infection is typically mild allowing insights for the nature history of infection to be garnered. The ferret model can be particularly useful for evaluating the upper airway, vascular, and inflammatory pathology of SARS-CoV-2 [15].

Rhesus macaques infected with SARS-COV-2 underwent a comprehensive transcriptomic evaluation of 14 tissues and organs collected 7-days from infection [16]. While most tissues had upregulated proinflammatory and angiogenesis pathways, the cerebral cortex displayed a unique transcript signature with marked up-regulation of coagulation proteins-prothrombin, factor XIII, von Willebrand factor, factor VIII, and plasminogen activator inhibitor (PAI)-1.

Eight non-human primates (11–16 years old) were inoculated with SARS-COV-2 via a multi-route mucosal or aerosol challenge [17]. Necropsies were performed 24–28 days from the time of initial inoculation and revealed the following features: neuro-inflammation, microthrombi, microhemorrhages, neuronal injury, and hypoxic-ischemic injury. There was minimal evidence of virus within the brain. Like human decedents with COVD-19 who underwent autopsy there were reactive astrocytes and microglia. The findings of hypoxic-ischemic injury raised the possibility of diffuse microvascular thrombosis with reduced perfusion. Because the brain is highly metabolic and requires aerobic metabolism of glucose to generate adenosine triphosphate (ATP) production, neuronal death or apoptosis would be the result of energy failure [17].

## Are there unique and distinct pathological and structural features of COVID-19 thrombosis?

### Non-COVID conditions

The foundation for determining a unique signature of COVID-19 thrombi begins with describing distinctive structure and composition of *arterial,* and *venous* thrombi based on aspirated and extracted specimens, and pulmonary emboli obtained at autopsy in patients with non-COVID-19 and non-viral infection conditions [18] (Table 2). Each of the distinguishing characteristics of *COVID-associated thrombosis* will be highlighted and illustrated in subsequent sections.

**Table 2:** The average volume fraction (%) of cellular and other components identified in arterial and venous thrombi and pulmonary embolism

Structures	Arterial thrombus	Venous thrombus	Pulmonary embolism
Total fibrin	43.3	35.0	41.2
Single fibers	11.2	12.0	24.7
Bundles	23.6	17.9	10.7
Sponge	8.5	5.1	5.8
Platelets	31.3	0.4	0.8
Microvesicles	5.0	0.7	3.5
White blood cells	1.5	0.3	5.4
Total RBC	17.1	63.4	48.6
Biconcave	1.7	2.1	0.0
Echinocytes	0.4	4.6	0.4
Compressed	15.0	56.7	48.2
Polyhedrocytes	13.2	37.5	39.4
Intermediate	1.5	19.2	8.3
Balloon-like	0.3	0.0	0.5
Space	1.8	0.2	0.5

Adapted from Chernysh IN. The distinctive structure and composition of arterial and venous thrombi and pulmonary emboli. *Sci Rep* 10, 5112 (2020)

### COVID-19

Genchi and colleagues characterized cerebral thrombi extracted from patients with COVID-19 and large vessel occlusion (LVO) ischemic strokes [19]. ACE2 was highly expressed in monocytes and macrophages and showed much higher expression than in controls (patients with LVO in the absence of COVID-19). There were no differences in erythrocytes, fibrin, neutrophil extracellular traps (NETs, see below), von Willebrand factor, platelets, and complement complex (C5b-9). Thrombi from COVID-19 patients did exhibit increased neutrophil density (MPO^+^ cells) and neutrophil-to-lymphocyte ratios-both had good discriminative ability to distinguish thrombi from patients with COVID-19 from those without COVID-19. Similar observations were made by Desilles and colleagues [20].

Khismattulin et al. reported on the structure and composition of pulmonary thrombi obtained from descendants with COVID-19 [21]. *Erythrocytes* were the prevailing type of cell with a deformed appearance secondary to platelet-mediated thrombus contraction. *Fibrin* was the second most common structural element and consisted of individual fibers, bundle fibers, and fibrin remnants. The preponderance of *immune cells* (CD3 + lymphocytes and CD20 + lymphocytes) was also a signature of thrombi among patients with COVID-19 [22].

### NETs structure and function

Upon activation, neutrophils release granule proteins and chromatin which together form extracellular fibers referred to as NETs (Neutrophil Extracellular Traps). The nuclei of neutrophils, upon stimulation, lose their shape and both the euchromatin and heterochromatin homogenize. The nuclear envelope and granule membranes disintegrate, allowing the mixing of NET components (chromatin and granule proteins). The NETs are subsequently released as the cell membrane disrupts. This cell death process is distinct from apoptosis and is dependent on reactive oxygen species [23].

While NETs have been described in thrombi from patients with or without COVID-19, an emerging question is whether there are either structural or functional differences. The contribution of NETs to the thrombosis phenotype and end-organ injury among patients with COVID-19 is an important area for consideration. Barnes and colleagues summarized their findings from an autopsy series and developed a working hypothesis to integrate pulmonary infection, cytokines, and thrombosis [24]. They observed the following pathological features in three descendants: neutrophil infiltration in pulmonary capillaries, acute capillaritis within fibrin deposition, extravasation of neutrophils into the alveolar space and neutrophilic mucositis.

### Nucleic acid cellular contents

The inflammatory environment that characterizes COVID-19 (summarized in greater detail in subsequent sections) has a direct effect on thrombus composition among patients with COVID-19. Cells that die because of acute injury typically swell and burst, releasing their contents in a highly detrimental inflammatory and prothrombotic response. The contents released include nucleic acids, chromatin, and histones (nucleosomes). By contrast, programmed cell death and apoptosis are characterized by cell shrinkage, condensing cytoskeleton collapse, nuclear envelope disassembly and fragmentation of nuclear DNA. In these instances, changes within the cell prompt rapid phagocytosis before there is significant leakage of cellular contents. In addition, the organic contents of the apoptotic cell are recycled by the phagocytosing cell(auto-phagocytosis) [25].

Wagner and colleagues studied freshly isolated neutrophils from patients with severe COVID-19 and found that they were primed for NETosis (40% of nuclei were positive for citrullinated histone H3). In addition, the neutrophils were forming inflammasomes such as NLRP3 as detected by ASC speck assembly, suggesting that inflammasome assembly preceded NETosis in affected COVID-19 patients [26].

Genchi et al. and others have identified a high density of NETs in thrombi obtained from patients with COVID-19 and arterial thrombosis (Lynch; unpublished observations)-a potential contributor to localized vascular injury, risk of recurring thrombosis, and autoimmune events that portend future plaque development [27, 28] Because only 20–25% of neutrophils are capable of releasing NETs, Yipp et al. report that tissue-specific low-density granulocytes co-localize with mononuclear cells [29] and act as a precursor to atherogenesis. Moreover, COVID-19 associated thrombosis may represent the penultimate example of inflammation preceding other cellular events-neutrophils being the first cells present at the site of thrombosis, even before platelets [30].

### Fibrinolytic resistance

The early experience with COVID-associated thrombosis, including occlusive coronary artery events suggested that impaired fibrinolytic response was a contributing feature. Weiss and colleagues reported a resistance to fibrinolysis even in the absence of significantly elevated fibrinogen levels as seen among patients with COVID-19 in the acute stage of illness [31]. Similar observations were made by Spiezia et.al [32]. While there are several potential mechanisms, the heightened inflammatory state and endothelial injury are likely contributors. Endothelial KLF2 mediates endothelium-dependent vascular homeostasis by differentially regulating endothelial genes, leading to an anti-inflammatory and antithrombotic endothelial surface with normal vasodilatory function. In contrast, the tumor suppressor p53 leads to inflammatory gene expression and impairs endothelium-dependent vasodilation, thus promoting endothelial dysfunction [33]. P53 and plasminogen activator inhibitor (PAI-1) expression are increased in COVID-19 [34].

The evidence suggests that the *COVID thrombus is immunoinflammatory* in nature and resistant to fibrinolysis, reflecting the end-result of immune events, primed neutrophils, and a highly inflammatory environment as described in the next section.

## Thrombus substrate and local environments

### Mechanistic considerations

The proclivity for thrombotic and thrombosis-associated events among persons with COVID-19, including anatomic sites of involvement and related phenotypes is based on many well-described mechanistic contributors. It is important to understand that there are organ-specific and tissue-specific factors that regulate both thrombosis and hemostasis that vary according to local and systemic conditions [35]. Similarly, these factors are dynamic and change from the time of initial infection, in response to homeostatic abnormalities, persisting triggers for thrombosis, or acquired conditions that are associated with immune thrombosis.

COVID-19 is associated with an inflammatory state and coagulopathy or acquired thrombophilia [36]. The terms thrombo-inflammation, immune-inflammation, and immune-thrombosis have been used to describe patients, primarily in the acute phase of infection but occasionally in the subacute phase or during recovery or in patients following even a mild infection. Early observations during the pandemic identified laboratory signatures for thrombosis, bleeding, and mortality particularly with very high D-dimer levels greater than 5 times the upper limit of normal and baseline platelet counts less than 150 × 10^9^ per liter [1].

### Neutrophil activation signature

Wang et al. [37] performed transcriptomics to determine dynamic differentially expressed genes (DDEG) in patients with COVID-19. Early and persistently activated neutrophils and coagulation protein pathways coupled with increased type I interferon signaling was consistently identified in patients who developed severe infections. Peripheral blood mononuclear cells PBMCs) [38] obtained from patients with COVID-19 reveal a marked increase in low intensity neutrophils, a population of cells recognized to be the primary source of NETs.

Middleton and colleagues [39] identified increased levels of MPO-DNA complexes in patients with COVID-19 and correlated directly and strongly with the severity of illness. Neutrophils obtained from critically ill patients displayed heightened NET formation in vitro that was attenuated by neutrophil NET inhibitory factor. Neutrophil activation signatures preceded the onset of critical illness and predicted mortality. Markers of neutrophil activation (RETN, LCN2, HGF, IL-8, G-CSF) were the most prominent discriminators of COVID-19 acuity [40].

Thierry and Roch [41] reviewed the potential association between dysregulated NET formation and COVID-19 with emphasis on uncontrolled inflammation, tissue damage and multi-organ system failure. The investigators raised an interesting scientific premise relating NET byproducts, specifically elastase, to accelerated virus entry, hemodynamic changes, and vasculitis. This line of thinking for COVID-19 and its most severe phenotypes also introduces targeted therapies such as DNase1, neutrophil elastase small molecule inhibitors-sivelestat, alvelestat and Bay-8550, inhibitors of cell free DNA re-entry like interleukin-26 and NET inhibitory peptides [42] into the dialogue of early pharmacological interventions that may offer both near-and long-term benefit in patients with COVID-19 (reviewed in Becker RC) [43].

Endothelial cell injury and dysfunction are associated with inflammation and coagulation that are vascular bed specific [44]. Case reports and case series, coupled with necropsy and post-mortem biopsies have documented micro- and macrovascular thrombosis involving the skin, dorsal lingual mucosa, brain, retina, lungs, heart, liver, kidneys, small intestine, colon, and upper and lower and extremities in patients with COVID-19. The overall frequency of thrombosis is highest in the pulmonary arteries and alveolar capillaries.

### Inflammasomes

Nucleotide-binding oligomerization domain-like receptor containing 3 (NLRP3) is an intracellular innate immune receptor that recognizes a diverse range of stimuli, including pathogens, damage cells and cellular debris (reviewed in Takahashi) [45]. NLRP3 activation promotes assembly of a large, multi-protein complex known as the NLRP3 inflammasome that in turn promotes inflammation, vascular injury, thrombosis, and cell death [46]. In addition, inflammasome activation causes pyroptotic macrophages to release tissue factor, increasing the propensity for thrombosis [47, 48].

### Circulating blood

Mustafa and colleagues [49] analyzed > 2 billion sequence reads of high-throughput transcriptome sequence data from 180 samples of patients with active SARS-CoV-2 infection or healthy controls collected from 6 studies and identified traces of SARS-CoV-2 RNA in peripheral blood mononuclear cells in a small number of cases. Whether this finding contributes to the early or later stages of disease, including a proclivity for inflammation or thrombosis has not yet been determined.

### Endotheliitis

The widescale expression of ACE2 receptors within endothelial cells represents a nidus for SARS-CoV-2 binding, membrane fusion and viral entry causing infection and attendant vascular injury and dysfunction. Engineered human blood vessel organoids can be infected with SARS-CoV-2 [50]. This can be blocked by human recombinant soluble ACE2. Varga et al. describe endothelial cell involvement across large and small, venous, and arterial vascular beds in descendants with COVID-19 [51]. Accumulation of inflammatory cells and viral inclusions by histology and electron microscopy, respectively, were identified within the endothelium of the heart, small bowel, kidneys, and lungs. In autopsy and surgical tissue specimens there was diffuse lymphocytic endotheliitis and apoptotic bodies. Maccio et al. performed a comprehensive analysis of cardiac autopsy tissue, including the coronary tree (main coronary arteries, epicardial arterioles/venules, epicardial capillaries) and epicardial nerves in 6 decedents with COVID-19 [52]. COVID-19 negative patients with cardiovascular disease (n = 3) and influenza A (n = 6) served as controls. COVID-19 positive decedents showed strong ACE2 / TMPRSS2 expression in capillaries and less in arterioles/venules. Epicardial *capillaries* had a prominent lympho-monocytic endotheliitis, which was less pronounced in arterioles/venules. The lymphocytic-monocytic infiltrate strongly expressed CD4, CD45, CD68- cellular receptors and antigens that signal immune activation. The main coronary arteries had only mild intimal inflammation. Pericardial/epicardial nerves displayed strong ACE2 expression and lympho-monocytic inflammation.

Khismattulin and colleagues examined tissue samples from the lung, kidney, brain, and heart of 45 patients with fatal COVID-19 [53]. Histologic, immunofluorescent, and both scanning, and transmission electron microscopy was performed. Inflammation and thrombosis were more pronounced in the lungs than the kidneys, brain, and the heart, and were most often associated with diffuse alveolar damage. Moreover, thrombi were most often found within microvascular beds and consisted of neutrophils, erythrocytes, platelets, fibrin, and virion-like particles. Histones and neutrophil extracellular traps (NETs) were also identified.

Magro and colleagues [54] reported the autopsy findings from five decedents with severe COVID-19 and adult respiratory distress syndrome (ARDS). They identified a pattern of tissue damage involving the lungs and integument consistent with complement-mediated microvascular injury. There was marked deposition of C5b-9, C4d and Mannan-binding lectin serine protease (MASP)-2, supporting a generalized activation of alternative and lectin-based pathways. Like other autopsy studies, they described pauci-inflammatory capillary injury with mural and luminal fibrin deposition. The term pauci (Latin-few) refers to the small amount of hypersensitivity upon immunofluorescent staining and involvement of small vessels. The skin lesions were characterized as pauci-inflammatory thrombogenic vasculopathy (reviewed in Becker RC) [55].

### Vascular endothelial glycocalyx integrity and dysfunction

The luminal surface of endothelial cells within arteries, veins and microvessels is coated with a thin (~ 500 nm) glycocalyx layer of plasma proteins, sulfated proteoglycans, glycoproteins and hyaluronan (reviewed in Weinbaum) [56]. Endothelial cell glycocalyx has several recognized functions, including maintaining vascular integrity, permeability, shear stress mechanotransduction, and inflammatory responses. Leukocytes traversing a small-caliber capillary can damage the glycocalyx. The transient deformation quickly corrects due to the elasticity of core proteins that behave like elastic fibers [56].

The properties of vascular endothelial glycocalyx layer change under inflammatory conditions. Cytokine-mediated activation of proteases partially degrades the layer permitting leukocyte rolling, tethering and recruitment [57]. An intact glycocalyx can regulate the degree of leukocyte capture, recruitment, and extravasation. Accordingly, a normal functioning glycocalyx is required to prevent inflammation and thrombosis.

### Infection—associated vasculitis

Another important question emerging from the COVID-19 experience is whether infection-associated vasculitis is unique to SARS-CoV-2 [58]. While the answer is no, this does not exclude unique and distinct features and temporal observations with the COVID-19 virus. Anti-neutrophil cytoplasmic antibody (ANCA) associated vasculitis following infection with mycobacterium species, Coccidiosis species, Rickettsia Ricketsii, Staphylococcus species, Epstein Barr virus, cytomegalovirus, and Dengue virus [59] have been reported. The typical time course from the onset of COVID-19 infection to the development of vasculitis is 3 months, and ~ 50% of patients have regression of vasculitis after resolution of the infection. The autoantibodies in ANCA-associated vasculitis are directed toward proteinase 3 (PR3) or myeloperoxidase expressed on the surface of neutrophils that either evade clearance, appear as cell fragments, or present in the form of extracellular traps (NETs) with exposure to auto-reactive T cells [60]. NETs can also cause direct endothelial cell injury (reviewed in Becker RC) [61].

Patients with pre-existing ANCA- associated vasculitis are more likely to have severe COVID-19 infections with a high mortality rate [62].

### Vasa vasorum injury

**Fig. 3:**
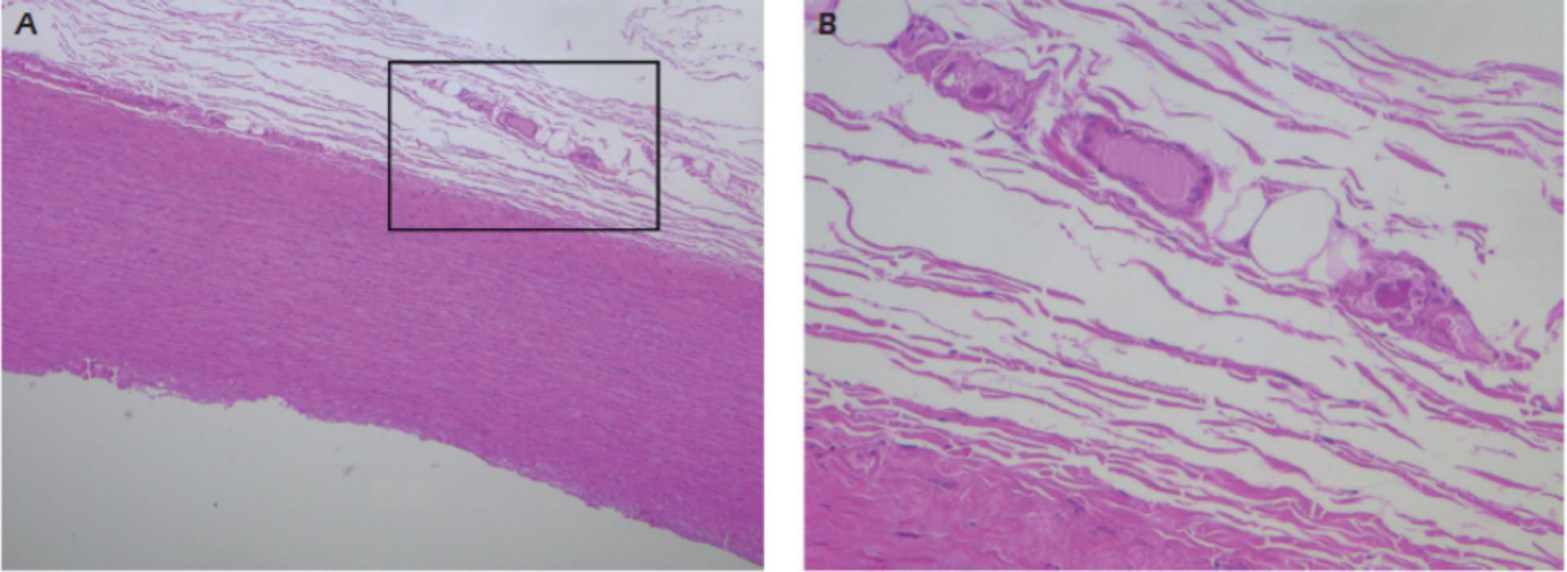
Vasa vasorum: full thickness of aortic wall at low power view (A original magnification 50 ×) showing occlusive micro-thromboses in the vasa vasorum at high power field (B original magnification 200 ×). (From Faa G. European Rev Med Pharm Sci 2022; 25:6439–6442. With permission)

The integrity of small, medium, and large caliber arteries depends on perfusion. Small and medium-sized arteries receive oxygen and other nutrients by diffusion. By contrast, large caliber vessels depend on their own circulation to a large degree. The latter is provided by the *vasa vasorum*. Moderate caliber arteries can also be highly dependent on the vasa vasorum when local conditions are a barrier to diffusion such as endothelial cell injury, glycocalyx disruption or dysfunction, atherosclerotic plaque and inflammatory states that involve the endothelium, intima and/or media [63]^,^ 64. Vasa Vasorum injury, disruption and resulting impaired response to endogenous vasoactive mediators have been reported in COVID-19. (Reviewed in Becker RC) [61, 65, 66] (Fig. 3).

### Spike protein endothelial cell infiltration

Endothelial cell and eccrine gland epithelium deposition of SP has been observed in cutaneous acral perniosis lesions (chilblains) [67]. Immunohistochemical staining shows SP alone with SP RNA, suggesting that cleaved SP may be a pathogenic factor in endotheliitis. Biopsies and staining reveal vasocentric and eccrinotropic T cell- and monocyte-derived CD11C + positive CD14 + and CD 123 + dendritic cell infiltrates. Typically, there is a marked expression of type 1 interferon inducible myxovirus resistance protein (MXA)-a marker of type 1 interferon signaling in tissues [54].

Magro et al. [68] described viral particles referred to as pseudo-virions docked deep within subcutaneous and other vascular beds. They classified the cutaneous features of COVID-19 into three categories 1. Complement-mediated thrombotic vascular injury 2. robust T cell type 1 interferon-driven inflammation and 3. humoral driven immune complex-mediated vasculitis cutaneous manifestations. Types 1, 2, and 3 are often associated with severe /critical, moderate, and mild acuity infection, respectively.

Intravenous injection of the S1 subunit in mice results in its localization in endothelial cells of mice brain microvessels showing colocalization with ACE2, caspase-3, IL-6, tumor necrosis factor α (TNF-α), and C5b-9, suggesting that the vascular pathology in COVID-19 can be induced by the S protein alone [69].

### Spike protein and thrombosis

Spike proteins, specifically the S1 subunit is an inflammagen and can cause structural changes to β- and ʎ- fibrin(ogen), complement, and prothrombin [70]. These structural changes make the proteins resistant to proteolytic cleavage and fibrinolysis. SARS-COV-2 spike protein provokes endothelial cell activation through α5 β1 and NFK B signaling [71] S1 also increases tissue factor expression, particularly upon co-activation with interferon-ʎ [72].

**Fig. 4:**
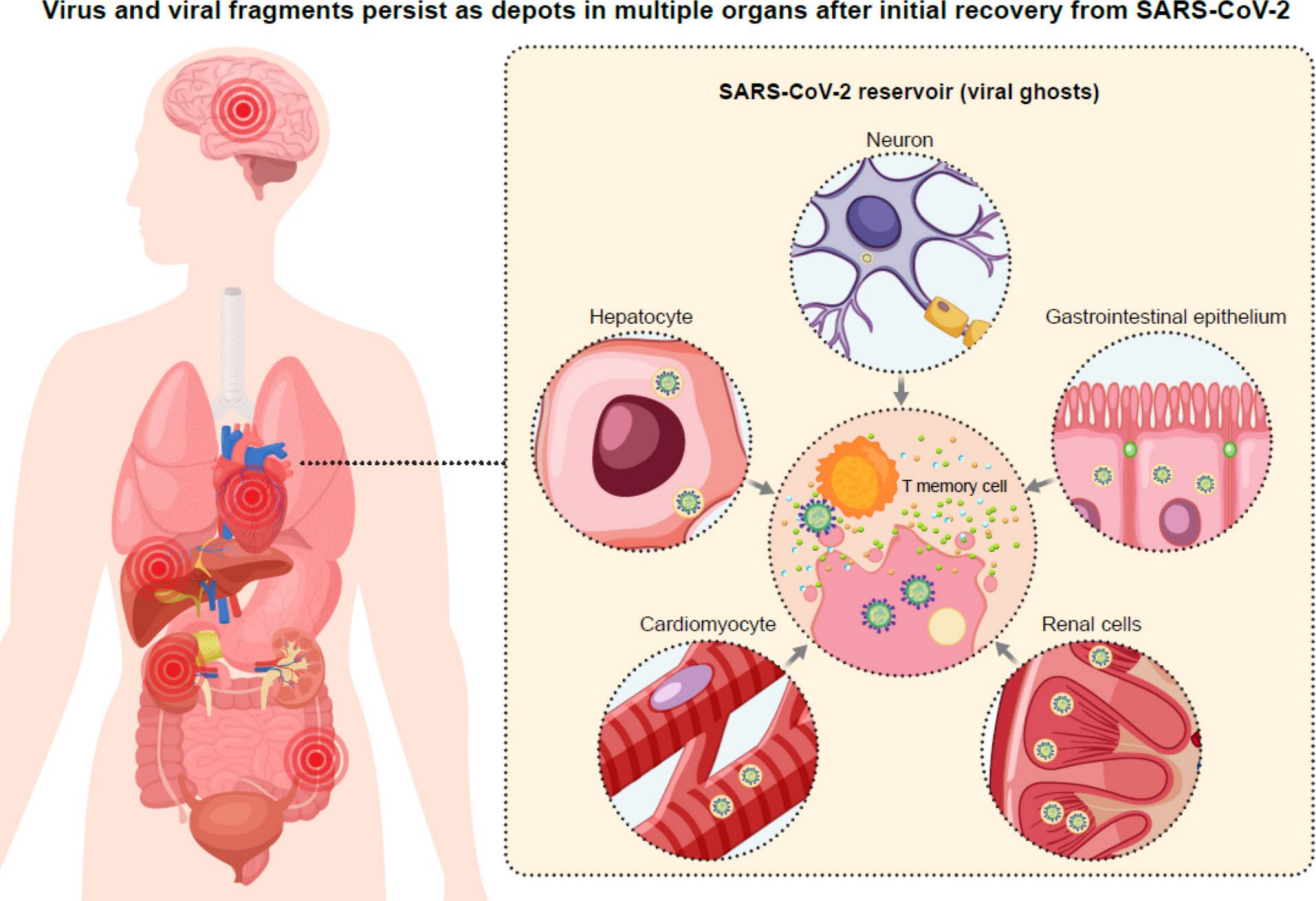
SARS-CoV-2 spike protein and thrombosis: the S1 subunit of SARS-CoV-2 has a major role in the thrombosis-phenotype that characterizes COVID-19. Activation of vascular endothelial cells, tissue factor expression, platelet activation, leukocyte recruitment, and neutrophil extracellular traps (NETs) formation are contributing mechanisms

The SARS-COV-2 spike protein activates microcirculatory EC causing complement (C3 and C5b-9) deposition, VWF-mediated platelet binding and aggregation, leukocyte recruitment, and down-regulation of thrombomodulin (responsible for modulating coagulation factors V and VIII and thrombin generation) [73] (Fig. 4).

Patterson et al. investigated the presence of SARS-COV-2 S1 protein in T-cell, B-cell, and monocyte subsets among patients with COVID-19 and post-COVID conditions [74]. The levels of intermediate and non-classical monocytes were elevated in patients compared to healthy controls. Some patients with severe COVID-19 and post-COVID conditions contained S1 protein- solely within non-classical monocytes. Fragmented SARS-COV-2 RNA was also detected in circulating peripheral mononuclear cells, but no full-length sequences were observed.

## The contributions of persistent virus or virus fragment depots, virus mutations and virus reactivation to COVID-associated thrombosis

**Fig. 5:**
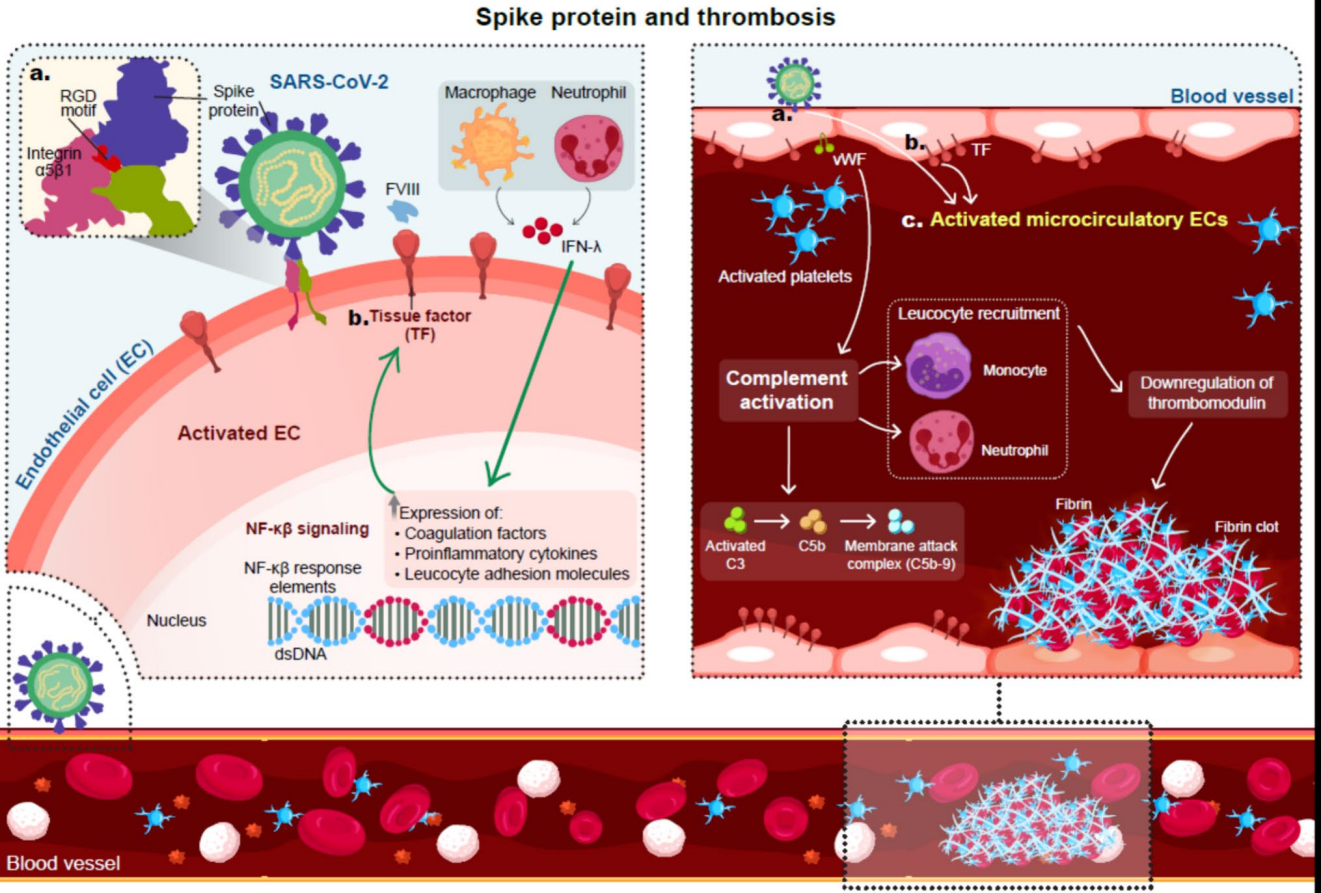
Virus and virus fragments in COVID-19: virus and viral fragments can be found in some patients following COVID-19. Reservoirs are believed to contribute to persistent immune activation and a systemic inflammatory state

Host cells expressing ACE2 receptors (and TMPRSS2) bind SARS-COV-2 followed by membrane fusion and internalization [75]. Intuitively, one might anticipate that tissue with the highest concentration or density of ACE2 receptors would serve as storage depot for SARS-CoV-2 [76]. Different amounts of replication-competent virus were detected in the culture media from the studied tissues. The highest viral load was measured in the lung (≈ 1.4 × 10^6^ copies/mL) and heart (≈ 1.9 × 10^6^ copies/mL) samples followed by the heart, gastrointestinal tract, kidneys, liver, and peripheral nerves (Fig. 5).

Evidence suggests that persistent and prolonged viral replication, inflammation, and autoimmune activity are potential mechanisms promoting thrombosis in the early and later phases of COVID-19 [77]. The persistence of the viral genome within a viral depot or reservoir in one or more organ systems is a potential explanation for persistent or recurring symptoms, where the virus or viral fragments create a nidus for continuous inflammation or an immune response from memory T cells.

### Periodontal pockets

Periodontal pockets are a main component of periodontitis, a common chronic inflammatory disease. The oral environment is ideal for harboring bacteria, viruses and biofilm that are in contact with the oral mucosa, bone, vascular system [78]. The clinical manifestations of harbored virus range from gingivostomatitis to herpes zoster, lymphoma, Kaposi’s sarcoma, and ulcerative stomatitis.

Extrapulmonary reservoirs of other coronaviruses include the brain (HCoV-22qe), liver (SARS-CoV), kidneys (endemic Balkan nephropathy virus), and gastrointestinal tract (HCoV-HKU1) [79]. These organ systems may potentially harbor structural, non-structural (NSP1, 3, 5, and 16), and accessory (ORF3a, 6 and 9) proteins interfering with immune responses and perpetuating symptoms [80].

### Nasopharynx

In a study conducted by Rodríguez-Grande et.al [81], RNA remnants purified from diagnostic nasopharyngeal specimens were used as the templates for RT-PCR-specific detection of SG E gene RNA (subgenomic (SG) viral RNA is expressed only in replicating viruses). As controls, they also detected viral genomic RNA for the E gene and/or a human housekeeping gene (RNase P). A total of 60 RT-PCR-positive cases with prolonged viral SARS-CoV-2 shedding (24 to 101 days) since the first diagnostic RT-PCR were sampled. SG viral RNA was detected in 12/60 (20%) of the persistent cases, 28 to 79 days after the onset of symptoms, suggesting the presence of actively replicating virus far beyond the initial diagnosis of COVID-19.

### Adipose tissue

Obesity is an independent risk factor and predictor of COVID-19 severity, hospitalization, ICU admission and mortality [82]. Early observations in decedents of severe COVID-19 suggested that adipose tissue may harbor SARS-CoV-2 signatures. Basolo and colleagues identified the SARS-CoV-2 genome in the adipose tissue of 56% of patients (13 of 23) [83], the virus nucleocapsid antigen in 1–5% of PCR positive adipocytes, and upregulation of the interferon α-pathway with associated leukocyte infiltration. Ryan et al. summarized several mechanisms underlying the observed proclivity for COVID-19 among obese populations, their worse clinical outcomes, heightened inflammation, impaired immune response, viral spread, and virus reservoirs [84]. Zickler et al. demonstrated SARS-CoV-2 infectivity in differentiated and lipid-laden adipocytes but not preadipocytes or immature precursors [85]. In non-human primates, SARS-CoV-2 is detected in subcutaneous adipose tissue within 7 days of infection and associated with reduced memory T cells [86].

### Monocytes

Patterson et al. reported a machine learning approach that identified the unique immunologic signature of individuals with PASC (post-acute sequalae of SARS-CoV-2 infection) [87]. The clinical relevance of monocyte activation in COVID-19 patients and the significance of these cells as a potential viral protein reservoir is supported by data reporting the presence of S1 protein within non-classical monocytes. Viral particles and/or viral proteins can enter monocyte subpopulations in distinct ways, and this appears to be regulated differently in individuals that will develop severe disease or post-COVID conditions that include thrombotic events. Considering their short circulating lifespan, viral protein-containing classic monocytes turn into intermediate and non-classical monocytes. Non classical monocytes have been proposed to act as custodians of vasculature by patrolling endothelial cell integrity [88].

In the case of post-COVID, the persistence of circulating S1-containing non-classical monocytes up to 15 months post infection indicates that certain conditions may exist to maintain this cell population. The S1 protein detected in patients with PASC appears to be retained from prior infection or phagocytosis of infected cells undergoing apoptosis and is not the result of persistent viral replication. By contrast, the observations support the hypothesis that an immune response to persistent viral antigens, specifically the S1 fragment of the spike protein eliciting an immune response marked by elevated inflammatory markers including IFN-γ, IL-6, IL-10, and IL-2, among others [74].

### Cell-free DNA

Short fragments of circulating DNA are the debris of dead cells from varied tissues and organ systems. Cheng and colleagues identified a signature of cfDNA in patients with COVID-19 that correlated with the WHO ordinal scale of disease progression in the acute phase of infection [89]. In addition to cfDNA from lung and liver sources, they identified cfDNA derived from red blood progenitor cells that might be the result of direct erythroblast involvement by the virus or the indirect consequence of hypoxemia and/or cytokine-mediated anemia that characterize severe COVID-19. Leppkes and coworkers demonstrated that, in severe COVID-19 patients, neutrophils were increased in the blood [90]. Plasma markers, including cfDNA were elevated in severe COVID-19 patients. cfDNA and aggregated NETs were detected in microvascular thrombi in the lungs and other organs of COVID-19 patients obtained by autopsy.

## Neuro-vasculopathy and thrombosis

The dominant organs and systems of involvement among patients with COVID-19 introduce several new constructs and fields of study that apply to an understanding of thrombosis. Among them include, but are not limited to, the central and peripheral nervous systems and their parallel circulatory systems as well as the autonomic nervous system (reviewed in Becker RC) [91].

### Sympathetic tone, inflammation and thrombosis

There is a time dependent variation in leukocyte adhesion within arteries, veins, arterioles, and venules that correlates with adhesion molecule expression and local sympathetic activation independent of blood vessel diameter [92]. Specifically, sympathetic innervation governs vascular inflammation and thrombosis following inflammatory insult. Because veins in the macro-and microvascular systems lack sympathetic fibers, the findings suggest that leukocyte accumulation and inflammation in the venous circulation is dependent on sympathetic events and β-2 adrenergic receptors in the arterial circulation.

### Autonomic dysfunction and inflammation

A higher in-hospital mortality has been associated with the early presence of neurological syndromes seen with COVID-19 [93]. Several key molecular factors are closely involved in stress-mediated dysregulation, including adrenaline, noradrenaline, and dopamine; peptide hormones and associated factors such as arginine vasopressin (AVP) and Ang II; and steroid hormones. This broad-based stress-mediated bioactive factor deregulation leads to activation of the sympathetic nervous system and a prothrombotic state [94].

## Platelets and the COVID thrombus

### Platelet activation and reactivity

It has been postulated that local platelet activation is an innate immune response to infected virus pathogen and promotes inflammation and coagulation during COVID-19 [95]. Elevated levels of platelet surface expression of P-selectin, soluble P-selectin and circulating microparticles released from platelets in patients with COVID-19 have been demonstrated [96].

Urinary 11-dehydro thromboxane B_2_ (u11-dh TxB_2_) has been used as a marker of platelet activation, platelet COX-1 activity, response to aspirin therapy and whole-body inflammatory state [97, 98]. Patients with COVID-19 have demonstrated markedly elevated levels of urinary 11-dehydro thromboxane B_2_ (u11-dh TxB_2_) as compared to patients with non-COVID-19 pneumonia. Patients with severe COVID-19 have greater u11-dh TxB2 as compared to those with less severe disease [99]. Higher platelet aggregation in response to α-thrombin was shown in patients with COVID-19 as compared to healthy subjects [98].

A possible underlying mechanism for the observation of platelet activation markers, heightened coagulation and elevated platelet reactivity has been attributed to the increased presence of immature platelet fraction in patients with COVID-19. The latter has been partially linked to disease severity [100].

### Direct effect of SARS-CoV-2 on platelets

In an in vitro experiment with blood samples from patients with COVID-19, platelet expression of ACE2 receptor and TMPRSS2 was associated with platelet activation, platelet aggregation, PAC-1 binding, P-selectin expression, and clot retraction [101]. Ribonucleic acid sequencing studies performed in bone marrow, lung tissue, and blood from COVID-19 patients demonstrated the presence of SARS-CoV-2 virions in megakaryocytes and platelets [102]. Elevated activation and major ultrastructural changes in platelets that were exposed to isolated S1 subunit was demonstrated in whole blood samples from healthy subjects. In addition, integrin α5β1, a fibronectin receptor that can interact with SARS-CoV-2 spike protein, has been shown to be expressed on the platelet and it was shown that α5β1 binding peptide, ATN-161, can block SAR-CoV-2 infection and to reduce platelet activation [103]. These observations indicate a potential direct interaction between platelets and SARS-COV-2.

The interaction between platelets and SARS-CoV-2 may explain thrombocytopenia, a common complication in patients with COVID-19. Thrombocytopenia has been associated with poor prognosis and high mortality in hospitalized COVID-19 patients [104]. A lower platelet count in patients with COVID-19 was shown to be associated with a three-fold increase in the risk of developing severe disease in a meta-analysis of 31 studies including 7613 patients [105]. Thrombocytopenia may be related to decreased platelet production, or increased platelet consumption [106].

## COVID-19 thrombosis phenotypes

### Venous thrombosis

Thrombosis involving the superficial and deep veins of the lower extremities, upper extremities, pelvic, splanchnic, pulmonary, and cerebral venous circulatory beds has been described in patients with COVID-19- primarily in the *hyperacute* (≤ 2 weeks) and *acute* (˃2–4 weeks) phases of infection [1]. There is a body of literature on venous thromboembolism (VTE) in the *subacute* phase as well (hospital discharge to 35-day follow-up) with an incidence of asymptomatic and fatal VTE of ~ 5% [107]. The risk of VTE is highest among patients with an elevated modified International Medical Prevention Registry on Venous Thromboembolism (IMPROVE) venous thromboembolism (VTE) score [108]-a 2–threefold increase of events in patients with a D-dimer level exceeding 500 ng/mL using local laboratory criteria or a score of 4 or more independent of the D-dimer level at the time of hospital discharge.

### Arterial thrombosis

While *Virchow’s triad* is a fundamental pathophysiologic construct for thrombosis in venous, arterial, and microvascular beds, there are heritable and acquired conditions that underscore an ability of thrombosis to occur without satisfying each of the three component parts -a process considered by some clinicians and investigators active in the field of thrombosis to represent *Virchow’s dyad* (COVID-19 Dyad)(reviewed in Becker) [109]. The primary determinant is a highly prothrombotic vascular surface on which platelet activation, coagulation protein assembly, thrombin generation and fibrin formation can occur, particularly with a concomitantly prothrombotic or prethrombotic circulatory state (discussed in a separate section). An *alteration in blood flow* may be represented by areas of non- laminar flow caused by inflamed vessel wall or edematous endothelial cells. The stasis of flow is not operational at least initially, but once even a small burden of thrombus is present localized Venturi or vortex flow patterns may amplify the thrombotic environment [110]. It is highly unlikely for medium and large vessel arterial thrombosis to occur in the absence of vascular inflammation and impaired fibrinolytic activity [111].

### Microvascular thrombosis and microangiopathy

The microvascular circulatory system consists of *arterioles, capillaries, and venules*. Arterioles have a primary role of regulating distribution of blood flow, while the capillaries represent the primary site of fluid and solute exchange, and the venules are the primary site of interaction with immune cells. The capillary beds do not hold a significant portion of the blood volume—estimated at less than 10% of the total volume—but have an enormous surface area for exchange (reviewed in Bray) [112]. Thrombosis has been defined mechanistically as the end-result of impaired biophysical properties of erythrocytes and leukocytes to include decreased deformability, heightened cell–cell interactions, soluble factors and perturbed or dysfunctional endothelial cells characterized by a loss of protective cell-surface anticoagulant, anti-platelet, fibrinolytic and anti-leukocyte proteins. Inflammation and both leukocyte and platelet-rich thrombi in a shear-stress dependent environment are the pathophysiological hallmarks of microvascular thrombosis [113].

In COVID-19, microvascular thrombi predominantly involve the *capillaries* with a signature of neutrophilic leukocytes, polyhedral erythrocytes, balloon-like platelets (from osmotic inflation) on the surface of inflamed endothelial cells and adjacent tissue injury with SARS-CoV-2 virions and microparticles- at times in large clusters [112]. Arteriolar thrombi are found in approximately 10% of autopsy samples. The features are readily distinguishable from those observed in patients with thrombotic microangiopathy [114] 24, 54.

### Concomitant macro- and microvascular thrombosis

Observations shared by pathologists around the world point to a very unique picture in decedents with COVID-19 infection: macro- and microvascular thrombosis with the former consisting of both red (erythrocytes, leukocytes, fibrin) and white (platelets and fibrin) thrombi and the latter platelet–fibrin thrombi in venules, arterioles and capillaries in all major organs including mesenteric fat, minimal evidence of microangiopathy, intravascular megakaryocytes, endocardial thrombi, viral particles in adipocytes and an unusual abundance of platelets in the spleen [115].

## An organ systems-based perspective of COVID thrombosis

The auto-antigen atlas developed by Wang and colleagues [37] provides insights into organ system involvement in COVID-19. Supramolecular fibril alterations were identified, including within molecular filament proteins. They included various isoforms of actin, actinin, collagen, filamin, fibronectin, fibulin, dynactin, dynein, lamin, myosin, nestin, nexilin, profilin, plectin, plastin, proteoglycan, septin, spectrin, talin, tropomyosin, tubulin, vinculin, and vimentin. These proteins are major components of the extracellular matrix, basement membrane, cell cytoskeleton, cytoskeletal motors, muscle filaments, and contractile motors of muscle cells.

The auto-antigen atlas also provided insights on dermatan sulfate (DS) affinity proteins. DS is the most potent among glycosaminoglycans in stimulating autoreactive B1 cells and autoantibody production. Platelet degranulation is found to be significantly associated with at least 18 altered proteins. DS-altered proteins are related to blood coagulation, platelet activation, platelet alpha granules, fibrinogen binding, fibrinogen complex, platelet plug formation, von Willebrand factor A-like domain superfamily, and platelet-derived growth factor binding. Collagen, which supports platelet adhesion and activation, and collagen biosynthesis and modifying enzymes are also among the COVID-altered proteins, e.g., collagen type VI trimer and type I trimer. Most of these altered proteins are known autoantigens, e.g., ALB, ANXA5, C1QBP, CALM1, CAPZB, COL1A1, COL1A2, COL6A1, FBLN1, FN1, PLEC, PPIB, THBS1, TLN1, TUBA4A, and YWHAZ  [37].

There are well described organ-specific factors that may determine the frequency of thrombosis,

even with a prothrombotic systemic phenotype that characterizes COVID-19 [116].

### Lungs

Scanning electron micrographs of large vessel pulmonary thrombi obtained in autopsy samples typically reveal a large volume or burden of erythrocytes and a lesser volume of fibrin. Intracellular borders are modest suggesting a highly compacted thrombus. In addition, there are deformed and compressed erythrocytes (polyhedrocytes) throughout both the central and peripheral vessels supporting a pivotal role of platelet-mediated contraction, retraction, and clot stiffening [117]. Studies of platelet contraction have shown that following contact with fibrin or fibrinogen, there is a rapid increase in the elastic modulus that can reach tenfold [118]. A unique feature of COVID-19 large vessel thrombosis is the high incidence of in situ events or primary pulmonary thrombosis rather than a more traditional path of thromboembolism [119].

### Pulmonary vein thrombosis

Pulmonary vein thrombosis most often arises following lung transplantation, lobectomy or in the setting of malignancy. There are cases reported in patients with COVID-19 [120]. Most patients are asymptomatic; however, nonspecific symptoms (such as dyspnea, cough, and hemoptysis are the result of pulmonary edema or infarction) can occur. Complications include pulmonary infarction, pulmonary edema, right ventricular failure, and less commonly, arterial embolism in the form of stroke and limb ischemia.

### Heart

#### Epicardial coronary arteries

There are many cardiovascular complications of COVID-19, including myocardial infarction that stems from an oxygen-supply mismatch (reviewed in Louis) [121]. Acute coronary artery thrombosis is also well described and ST segment elevation MI in patients with COVID-19 portends a higher risk of death than in those without the infection and its multiorgan system involvement [122]. While initial hesitancy to expose catheterization laboratory staff to infected patients and delays both in patients seeking medical care and initial assessment led to an increased use of fibrinolytic therapy, propensity matching drew similar conclusions of worse outcomes in patients with COVID-19 [123].

Patients with underlying cardiovascular risk factors and existing coronary artery disease are at heightened risk for poor outcomes following COVID-19 and associated MI, however, the pathophysiology and thrombus characteristics have unique features. First, patients with coronary artery thrombosis may have minimal atherosclerotic plaque and no evidence of plaque rupture or erosion. Second, the thrombus itself contains a high density of NETs. Third, optimal treatment may include extraction thrombectomy rather than stent placement [124]. Multiple vessel coronary artery thrombosis has also been reported [125].

#### Heart chambers

Intracardiac thrombosis can occur in COVID-19. In most cases, there is either a preceding cardiomyopathy, pulmonary embolism with right heart dilation, acute MI with involvement of the left ventricle and/or right ventricle or myocarditis [126]. While ventricular thrombosis is most common, right atrial thrombosis can also occur-most often because of thrombus in transit from a deep vein nidus or adjacent central venous catheter or canula for patients on ECMO (extracorporeal membrane oxygenation) or those receiving either hemodialysis or continuous renal replacement therapy [127]. Cerebral and systemic thromboembolism are not considered rare complications of cardiac chamber thrombosis.

#### Microvascular disease

Coronary microvascular dysfunction (MVD) is common among patients with COVID-19 in the *hyperacute* and *acute* phases of illness [128]. Coronary flow velocity is impaired-particularly among patients with severe infection and multi-organ system injury with dysfunction. Biomarkers of inflammation, myocardial injury, and fibrin degradation correlate with the hyperemic coronary flow velocity. Global myocardial perfusion reserve assessed by cardiac magnetic resonance response cine and late gadolinium enhancement, as well as velocity encoded phase contrast imaging of coronary sinus flow at rest and following vasodilation with intravenous regadenoson is reduced by 35% in patients with COVID-19 and persisting dyspnea on exertion [129]. Thrombolysis in myocardial infarction (TIMI) frame counts are also higher in this patient population [130]. While the mechanism(s) underlying MVD in COVID-19 are multifactorial Endothelial cell inflammation, microvascular thrombosis, and microvasculopathy are common themes [131].

#### Aorta

The large caliber and high laminar flow conditions of the aorta make it an uncommon site for thrombosis. Conditions in which aortic thrombosis occur include the following: dissection, ulceration, aneurysms, plaque disruption, solid organ malignancy, myeloproliferative disorders, non-biological materials such as endografts, Takayasu’s aortitis, Behcet’s disease, temporal arteritis with great vessel involvement, IgG4-related disease, and trauma. The common theme for each is inflammation in a highly prothrombotic environment that may exist both locally and systemically. Petrov and colleagues [132] summarized an existing literature on aortic thrombus that included 56 patients with COVID-19. Most patients had no prior history of aortic disease. Thrombosis was described in all parts of the aorta (ascending, transverse, and descending) including multiple sites, detected approximately 10 days from symptom onset and was frequently associated with peripheral thromboembolism. The short- term mortality rate was 30.4%.

#### Hepatic, portal, and mesenteric system

Hepatic artery thrombosis has been reported in patients with COVID-19 and typically presents with acute abdominal pain or markers of liver injury when occurring in isolation or with hepatic-portal-mesenteric axis thrombosis [133]. Like renal artery thrombosis (discussed in a subsequent section), hepatic artery thrombosis can involve either native or transplanted organs [134]. Portal vein thrombosis is a more common thrombotic complication in COVID-19 [135]. iT occurs either in the presence or absence of pre-existing cirrhosis [136] and can involve either the extrahepatic or intra-hepatic veins. Concomitant splanchnic or mesenteric vein thrombosis has also been reported [137].

Superior mesenteric artery and superior mesenteric vein occlusive thrombosis have been reported in COVID-19 presenting with acute abdominal pain. As with other thrombosis-related complications, the thrombotic event may precede a laboratory confirmed diagnosis-particularly in patients with mild constitutional symptoms [138]. Celiac artery thrombosis with splenic infarction can also occur [139].

#### Peripheral artery system

Several studies have described acute thrombosis-related limb ischemia in critically ill patients with COVID-19 [140]. Patients with less severe infection, including those who either did not require initial medical attention or hospitalization have also been reported with acute limb ischemia involving the upper or lower extremities [141].

### Brain

#### Large vessel occlusion stoke

COVID-19 was diagnosed in greater than half of all patients with large vessel occlusion stroke during the peak of the pandemic [142]. There were differing patient characteristics that warrant consideration. Those with stroke and COVID-19 were younger (by 10 years), more often male and more often non-white when compared to patients without COVID-19. Among all patients with COVID-19 and stroke, nearly one quarter had large vessel occlusion—most often involving the middle cerebral artery and its M1 and M2 segments. Ten percent of patients had multifocal large vessel occlusion. Arteritis and large vessel occlusion stroke have been reported in the acute and subacute phases of infection [143].

Thrombus characteristics were as follows leukocytes, platelets, and erythrocytes with minimal fibrin. A large proportion of leukocytes were neutrophils co-localized with DNA myeloperoxidase and H3Cit (NETS) [144]. While SARS-COV-2 has been isolated from thrombi [19], this is not a common finding.

#### Cerebral veins

COVID-19 associated cerebral venous sinus thrombosis is well discussed in the medical literature and should be considered in patients with severe headaches either with or without mental status changes. In many cases, multiple dural venous sinuses are involved and involvement of the deep venous sinuses occurs in nearly 50% of patients [145].

#### Pulmonary venule thrombosis and stroke

A novel mechanism for acute stroke has been described in patients with COVID-19 wherein pulmonary vein thrombus can extend into the left atrium and subsequently embolize to the brain [146]. Appreciation of this mechanism requires an understanding of the tricompartmental model of lung parenchyma oxygenation (the alveolus, the bronchial artery, and the pulmonary artery), each of which is compromised in COVID-19. Of these 3 sources, the bronchial artery plays a crucial role in COVID-19 stroke because the unique collaterals from bronchial artery to pulmonary vein which exist under normal physiological conditions (and maintain venous patency when the pulmonary artery is blocked by embolus) are occluded, leading to venular thrombosis in the presence of hypercoagulability. Dislodgement of clots from this source may account for many cases of large vessel occlusive stroke in COVID-19.

#### Retina

A large cohort study from an integrated health care system [147] identified an increased incidence of retinal vein occlusion among the participants with COVID-19 in the preceding 6 months. Retinal artery embolism has also been reported [148].

#### Kidney

Renal artery and vein thrombosis has been reported in COVID-19 [149]. Thrombotic micro-angiopathy involving the glomeruli, arterioles, and venules associated with cortical infarction has also been reported [150]. Renal artery thrombosis is most often unilateral but can be bilateral and while concomitant aortic thrombosis is a more common nidus, isolated renal artery thrombosis in native or a transplanted kidney is well described [151, 152].

#### Olfactory system

A multi-center post-mortem cohort study of 23 decedents with COVID-19 was conducted by Ho et al. [153]. Olfactory tissue from patients compared to controls (with non-infectious disease) was found to have several distinct features: a higher axonal pathology score, decreased axon density, endothelial cell injury, and a higher vasculopathy score.

#### Genitourinary and reproductive systems

While there has been discussion about male and female reproduction during the pandemic and the potential role of microvascular thrombosis [154], a consensus has not been reached. Ovarian vein thrombosis has been reported in COVID-19 [155]. Superficial thrombosis of the penile dorsal vein has been described [156].

#### Musculoskeletal system

Patients, particularly those in the *recovery* phase of COVID-19 or with post-COVID conditions often have musculoskeletal pain, stiffness, and occasionally weakness. The combination of a viral illness associated with microvascular thrombosis and frequent use of corticosteroids predisposes to osteonecrosis of the femoral head [157] or avascular necrosis. Asymmetrical gait patterns are observed commonly in patients who have made a full recovery [158]. Sarcopenia is common, particularly in patients with serious illness and in older people with preceding periods of inactivity during quarantine [159]. In addition, dysregulation of muscle protein synthesis and breakdown has been described in COVID-19, stemming from heightened inflammation, oxidative stress, use of corticosteroids, and treatment with antiviral drugs. Using the gene expression omnibus (GEO) database Cantu et.al [160] identified down-regulation of several skeletal muscle-related genes, including FOX01, Malat1, TIN and CXXC5 in patients with COVID-19.

#### Integument

The frequency of cutaneous manifestations of COVID-19 is approximately 10% with features ranging from urticaria to erythema pernio, erythema multiforme, maculopapular squamous erythema, livedo reticularis, purpuric vasculitis and Chibiusa vesicular eruptions [161].

Arteriolar thrombosis is common among critically ill patients and are complement C5-C9 deposition, tissue factor expression, SARS-COV-2 virus, and spike protein. Microvascular alterations include microhemorrhage, capillary thrombosis and neo angiogenesis [162], palpable purpura hemorrhagica bullae, acrocyanosis and skin necrosis have been reported [163].

## A temporal perspective of COVID thrombosis

### Hyperacute phase

There is a direct relationship between thrombosis involving the microvascular and macrovascular circulatory systems and COVID-19-related morbidity and mortality [22]. While the latter vascular system can be either arterial or venous and undoubtedly contributes to disease acuity, the former is multi-organ system associated with accompanying multi-organ failure. Despite a well-described COVID-19 coagulopathy that characterizes the *hyperacute* and *acute* phases of illness, treatment with traditional anticoagulant and antithrombotic therapies has not yielded a clear benefit in randomized clinical trials [164]. By contrast, non-critically ill patients characterized as thrombosis-prone but with events proportionally greater in the macrovascular rather than the microvascular circulatory systems do derive benefit from anticoagulant therapy [165].

### Prevention and treatment of microvascular thrombosis

The treatment of microvascular thrombosis, particularly in the setting of severe infection has traditionally focused on treating the underlying cause [75]. The same approach currently applies to COVID-19, but pathophysiology-directed therapies must be considered to complement anti-viral and anti-inflammatory approaches [166]. The challenge lies in the complexity of the microvasculature itself, including its endothelium, glycocalyx, and regulatory mechanisms that typically prevent thrombosis, and maintain steric and change-dependent barriers and both flow and mechanoreceptor dynamics (Summarized in Bray) [112]. Moreover, there are organ and organ system-specific characteristics of microvasculature that significantly impact preferred targets and anticipated responses to inhibition [167].

Determining specific mechanism(s) for COVID-19 associated microvascular thrombosis is the key to effective prevention and treatment. As a starting point, establishing similarities and likenesses to other conditions known to cause microvascular thrombosis and microangiopathies is important- particularly if there are tested, safe and effective therapies. One example is catastrophic antiphospholipid syndrome (CAPS) that presents with sudden and widespread microvascular thrombosis and associated kidney, lung, liver, heart, and brain injury [168]. Infection is the most common trigger of CAPS and peripheral blood tests demonstrate anticardiolipin antibodies, anti-β2 glycoprotein 1 antibodies, and a positive lupus anticoagulant screen. Treatment consists of unfractionated heparin, high-dose steroids, and plasma exchange transfusions. IVIg has also been used successfully [169].

### Anticoagulant therapy to prevent COVID-19 associated thrombosis: evidence from randomized clinical trials

The pathobiological underpinnings of severe COVID-19 and coagulopathy are well described [109]. The clinical trials of anticoagulant therapy conducted to date offer important insights.

The large, randomized clinical trials (REMAP-CAP, ACTIV4) [164] and a more modestly sized COVID-PACT [170], offer a level of consistency and clinical guidance for anticoagulant therapy in COVID-19 among patients requiring ICU-level care. Therapeutic anticoagulation with LMWH or UFH reduced VTE, increased non-fatal bleeding, but did not lower mortality. Among moderate acuity hospitalized patients [171], therapeutic anticoagulation reduced the likelihood of worsening clinical status and increased organ support free days. These are the primary messages for practicing clinicians.

Exploratory analysis of a multiplatform adaptive RCT of therapeutic-dose heparin vs usual care pharmacologic thromboprophylaxis was conducted in 3,320 patients hospitalized for COVID-19 enrolled in North America, South America, Europe, Asia, and Australia between April 2020 and January 2021 [164]. Heterogeneity of treatment effect was assessed 3 ways: using (1) conventional subgroup analyses of baseline characteristics, (2) a multivariable outcome prediction model (risk-based approach), and (3) a multivariable causal forest model (effect-based approach). Analyses primarily used Bayesian statistics, consistent with the original trial. In the overall multiplatform RCT population, therapeutic-dose heparin was not associated with an increase in organ support–free days (median value for the posterior distribution of the OR, 1.05; 95%credible interval, 0.91–1.22). In conventional subgroup analyses, the effect of therapeutic-dose heparin on organ support–free days differed between patients requiring organ support at baseline or not (median OR, 0.85 vs 1.30; posterior probability of difference in OR, 99.8%), between females and males (median OR, 0.87 vs 1.16; posterior probability of difference in OR, 96.4%), and between patients with lower body mass index (BMI < 30) vs higher BMI groups (BMI ≥ 30; posterior probability of difference in ORs > 90%for all comparisons). In risk-based analysis, patients at lowest risk of poor outcome had the highest propensity for benefit from heparin (lowest risk decile: posterior probability of OR > 1, 92%) while those at highest risk were most likely to be harmed (highest risk decile: posterior probability of OR < 1.87%.

How do the findings of ANTI-COVID [172] add to a large body of literature on the topic and inform clinical practice? They align with prior observations made throughout the pandemic that patients with COVID-19 who are hypoxemic and require hospitalization are at heightened risk for VTE, including catheter-associated thrombosis and that increasing the intensity of anticoagulation reduces the risk. Could high-dose prophylactic anticoagulation represent the sweet spot for efficacy and safety?

A lingering question is why heparin-based anticoagulant therapy at any intensity does not lower mortality? Multiple organ dysfunction syndrome (MODS) or multi-organ system failure (MOSF) is the most common cause of death in patients with COVID-19, followed by secondary bacterial infections, including ventilator-associated pneumonia, refractory hypoxemia, and macrovascular ischemic events (venous or arterial). While inflammation, dysregulated immune responses, and thrombosis (micro-and macro-vascular), collectively referred to as COVID-19 coagulopathy and endotheliopathy [61], represents a common pathobiological underpinning for poor outcomes, mechanistic details are lacking, and traditional anticoagulant therapy based on the available data does not address the responsible targets or upstream pathways to a necessary or sufficient degree to favorably alter mortality.

## Acute phase

The incidence of VTE among persons with COVID-19 who do not require hospitalization is increased compared to propensity-matched controls (50.9 versus 2.37 per 1000 patient years respectively; HR 214: 95%CI 12.63–363) [173]. Older age, male sex, obesity, BMI˃40 kg/m^2^, Black race, and inherited thrombophilia are independently associated with an increased risk. Vaccination was associated with a substantial reduction in risk (HR 5.95, 95% CI 1.82–19.5, interaction P = 0.02) [174].

## Subacute phase

A clear understanding of thrombotic risk in COVID-19 requires defining events that occur after the *acute* phase of the illness. Moreover, one must be able to distinguish primary events from secondary events- the result of inadequate attention and prioritization of chronic conditions and non-COVID illnesses or conditions that predispose to thrombosis. Several examples include the treatment of hypertension, diabetes mellitus, hyperlipidemia, cancer and cancer screening and secondary prevention following a prior myocardial infarction, stroke, or coronary artery revascularization [175].

Patients with peripheral artery thrombotic events in the setting of COVID-19, including those treated with open surgical intervention, endovascular procedures or anticoagulation alone had patency rates and limb salvage rates of 50–60% and 89.2%, respectively [176].

## Convalescent and chronic phase

Ambulatory patients with COVID-19 participating in the UK Biobank were evaluated for incident VTE. In 18 818 outpatients with COVID-19 (10, 580 women [56.2%]; mean [SD] age,64.3 [8.0] years) and 93,179 matched uninfected participants (52 177 women [56.0%];mean [SD] age, 64.3 [7.9] years), the infection was associated with an increased risk of VTE at 30 days (incidence rate of 50.99 and 2.37 per 1000 person-years for infected and uninfected people, respectively; HR, 21.42; 95%CI, 12.63–36.31). Older age, male sex, and obesity were independently associated with higher risk, with adjusted HRs of 1.87 (95%CI, 1.50–2.33) per 10 years, 1.69 (95%CI, 1.30–2.19), and 1.83 (95%CI, 1.28–2.61), respectively. An inherited thrombophilia was associated with a HR of 2.05 (95%CI, 1.15–3.66) for post–COVID-19 VTE. The risk was substantially less among patients who had been fully vaccinated and experienced breakthrough infection [177].

## Post-discharge phase

Cohort studies of patients with COVID-19 who are discharged from the hospital and followed closely over time provide an important means to determine longitudinal outcomes and the incidence of thrombosis [178]. In a cohort study of 2,832 patients hospitalized with COVID-19 (Li), 36 (1.3%) had post-discharge VTE and 15 (0.5%) had arterial events during the 90-day follow-up period. Patients with prior VTE, peak D-dimer levels ˃3.0 µg/ml and elevated CRP (> 10 mg/dl) at the time of discharge were at highest risk for VTE.

An open-label, multicenter, randomized trial was conducted at 14 centers in Brazil [107]. Patients hospitalized with COVID-19 at increased risk for VTE (International Medical Prevention Registry on Venous Thromboembolism [IMPROVE] venous thromboembolism [VTE] score of ≥ 4 or 2–3) [179]with a D-dimer > 500 ng/mL were randomly assigned (1:1) to receive, at hospital discharge, rivaroxaban 10 mg/day or no anticoagulation for 35 days. The primary efficacy outcome in an intention-to-treat analysis was a composite of symptomatic or fatal VTE, asymptomatic VTE on bilateral lower-limb venous ultrasound and CT pulmonary angiogram, symptomatic arterial thromboembolism, and cardiovascular death at day 35. A total of 320 patients were enrolled. The primary efficacy outcome occurred in five (3%) of 159 patients assigned to rivaroxaban and 15 (9%) of 159 patients assigned to no anticoagulation (relative risk 0·33, 95% CI 0·12–0·90; p = 0·0293). No major bleeding occurred in either study group.

Using the National Healthcare Database of the United States Department of Veterans Affairs Al-Aly et. Al systematically determined 6-month incident thrombotic events in patients with COVID-19 who survived for at least 30 days after diagnosis [180]. An excess burden of incident pulmonary embolism (HR 2.63, 95% CI 2.25–2.92) was observed. Adjusted hazard ratios for stroke and overall thromboembolism were also increased. In each instance, the risk for future events following initial infection was increased among persons testing positive, requiring hospitalization, and needing ICU-level care. Future risk was greatest in high acuity patients.

Xie and colleagues [173] used the VA database to construct a cohort of 153,760 individuals with COVID-19, as well as two sets of controls with 5,637,647 (contemporary controls) and 5,859,411 (historical controls). The duration of follow-up was approximately 1-year. The hazard ratios for myocardial infarction, pulmonary embolism, deep vein thrombosis, and superficial vein thrombosis were 1.63, 2.93, 2.09, and 1.95 respectively. The risk of a composite of thrombotic disorders was 2.39. Considered from a population health perspective, the findings can be summarized as follows:

- 9.88 incidents of thromboembolic disorders, including 5.47 incidents of pulmonary embolism and 4.18 incidents of deep vein thrombosis per 1000 patient-years.
- 23.48 incidents of major adverse cardiovascular events, including MI, stroke, and all-cause mortality per 1000 patient- years.

The Northwell Health Registry investigators performed a prospectively designed registry of hospitalized patients with COVID-19 who survived discharge [181]. Ninety-day outcomes were determined in 4906 patients. The rate of VTE and arterial thromboembolism (ATE) were 1.55% and 1.71%, respectively. The composite outcome of VTE, ATE, and all-cause mortality was 7.13%. The risk was increased among patients with advanced age (OR 3.66, 95% CI-2.84–4.71) and those with an IMPROVE-DD VTE Risk Scores ≥ 4 (OR 1.51; 95% CI -1.06–2.14).

A cohort study of patients with COVID-19 hospitalized at the Henry Ford Health System reported new onset VTE and ATE at 90 days from discharge [178]. A total of 2832 patients were followed. Thirty-six patients (1.3%) and 15 patients (0.5%) were diagnosed with VTE and ATE, respectively. Patients with prior VTE (OR 3.24; 95% CI, 1.34–7.86) were at greater risk.

## Antiplatelet therapy studies

The large body of evidence demonstrating thrombotic complications in COVID-19 associated with hypercoagulability and less so with platelet activation has encouraged investigators to utilize antiplatelet and anticoagulant agents to reduce the risk of thrombotic events [182]. A prospective observational study of hospitalized patients with COVID-19 reported lower u11-dh TxB_2_ levels in patients on aspirin therapy than patients not on aspirin therapy (p = 0.003) [183]. In another observational cohort study of adult patients with COVID-19, aspirin use (mostly 81 mg daily dose) at least 7 days before hospitalization or within 24 h of hospitalization compared to non-aspirin use was associated with lower rates of mechanical ventilation (36% vs. 48%,) and intensive care unit (ICU) admission (39% vs. 51%) [184] These initial observations supported the hypothesis that administration of aspirin with its antiinflammation, antithrombosis, and antiviral properties provides an effective adjunctive therapeutic option in patients with COVID-19 [182].

In a study of American Veterans with COVID-19, preexisting aspirin prescription was associated with a significant decrease in overall mortality at 14 days and at 30 days compared to patients who were not treated with aspirin [185]. Similarly, in a propensity score-matched observational study of COVID-19 patients (n = 638), in-hospital aspirin therapy compared to no antiplatelet therapy was associated with a significantly lower cumulative incidence of in-hospital death (HR, 0.522) [186]. In another retrospective population-based cross-sectional investigation, patients who were on aspirin for primary prevention had a lower rate of COVID-19 as compared to aspirin non-users (OR, 0.71; p = 0.04), and a shorter clinical duration of COVID-19 (19.8 ± 7.8 vs. 21.9 ± 7.9 days, p = 0.045) [187].

In a propensity score-matched cohorts of patients including 6,781 patients on prehospital antiplatelet therapy (84% aspirin and clopidogrel 8.2%) and 10,566 patients not on-antiplatelet therapy groups, significantly lower in-hospital mortality was reported in patients receiving prehospital antiplatelet therapy (HR: 0.81, p < 0.005) [188]. In an observational cohort of 112,269 patients from the National Institute of Health's National COVID Cohort Collaborative (N3C), early aspirin use was associated with significantly lower 28-day in-hospital mortality (OR = 0.85; P < 0.001) and pulmonary embolism (OR = 0.71; P = 0.004), but not deep vein thrombosis, gastrointestinal hemorrhage cerebral hemorrhage, or blood transfusion [188]. An analysis of 27 studies, including a pooled meta-analysis of 23 studies and 4 randomized clinical trials were narratively synthesized [189]. Based on 23 observational studies of 87,824 COVID-19 patients, antiplatelet treatment favored a lower risk of mortality [odds ratio (OR) 0.72, 95% confidence interval (CI) 0.61–0.85; *I*^2^ = 87.0%, *P* < 0.01]. The narrative synthesis of RCTs did not support adding antiplatelet therapy to the standard care, regardless of the baseline illness severity and concomitant anticoagulation intensity.

In the international RECOVERY trial of 14,892 hospitalized COVID-19 patients who were treated with 150 mg aspirin as compared to usual care there was a similar rate of death in 28 days (17% vs. 17%), no reduction in the risk of progression to the composite endpoint of invasive mechanical ventilation or death in patients not on invasive mechanical ventilation at baseline (21% vs. 22%), a 0.6% absolute reduction in thromboembolic events (4.6% vs. 5.3%) and a 0.6% increase in major bleeding (1.6% vs. 1.0%) [190]. In the ACTIV-4a clinical trial, 562 non-critically ill patients hospitalized for COVID-19 were to a therapeutic dose of heparin plus a P2Y_12_ inhibitor with ticagrelor as a preferred P2Y_12_ inhibitor or a therapeutic dose of heparin only (usual care) for 14 days or until hospital discharge, whichever was sooner. In this trial, the combination of a therapeutic dose of heparin plus a P2Y_12_ inhibitor compared with usual care was associated with similar days free of respiratory or cardiovascular organ support up to day 21 of the index hospitalization (21 days in each group, primary endpoint) and there was no decrease in mortality or the rate of thrombotic events with heparin plus P2Y_12_ receptor inhibitor strategy [191]. In the subsequent ACTIVE-4B trial, 657 symptomatic outpatients with COVID-19 were randomized to aspirin (81 mg orally once daily), prophylactic-dose apixaban (2.5 mg orally twice daily), therapeutic-dose apixaban (5 mg orally twice daily), or placebo (n = 164) for 45 days in a 1:1:1:1 ratio. The primary composite end of all-cause mortality, symptomatic venous or arterial thromboembolism, myocardial infarction, stroke, or hospitalization for cardiovascular or pulmonary cause occurred in 1 patient (0.7%) in the aspirin group, 1 patient (0.7%) in the 2.5-mg apixaban group, 2 patients (1.4%) in the 5-mg apixaban group, and 1 patient (0.7%) in the placebo group. Thus, there was no benefit of aspirin or apixaban compared with placebo in hospitalized COVID-19 patients [192]. In the REMAP-CAP international adaptive platform trial, 1557 critically ill adult patients with COVID-19 were randomized to receive either open-label aspirin, a P2Y12 inhibitor, or no antiplatelet therapy. In this trial, antiplatelet therapy (aspirin, and P2Y_12_ antagonists) vs. no antiplatelet therapy in addition to a prophylactic dose of anticoagulation was associated with similar of primary endpoint of organ support-free days (days alive and free of intensive care unit-based respiratory or cardiovascular organ support) within 21 days (7 days in each group) but there was an increased risk of major bleeding with antiplatelet therapy group (2.1% and 0.4%) [193]. In a systematic review and meta-analysis, although 23 observational trials of 87,824 patients indicated a lower risk of mortality associated with antiplatelet therapy, the narrative synthesis of the above mentioned 4 randomized trials did not confirm similar benefit [194].

Based on the results of the REVOVERY and ACTIV-4a trials, the recent International Society on Thrombosis and Hemostasis (ISTH) guidelines stated that “in non-hospitalized patients with symptomatic COVID-19, initiation of antiplatelet therapy is not effective to reduce the risk of hospitalization, arterial or venous thrombosis, or mortality (Class 3 no benefit, LoE:B-R) and based on the results of RECOVERY and REMAP-CAP trials, it stated “ in select critically ill patients hospitalized for COVID‐19, add on treatment with an antiplatelet agent to prophylactic dose LMWH/UFH is not well established but might be considered to reduce mortality” [195].

In summary, the direct role of platelets in COVID-19 remains controversial. Direct evidence from autopsy studies indicated the presence of fibrin, erythrocytes, and neutrophils with NETs in pulmonary microthrombi with a lesser presence of platelets. In the systemic circulation, elevated levels of platelet activation markers, platelet-neutrophil aggregates and NETs have been reported. Thrombocytopenia, elevated levels of immature platelet fraction and less evidence for heightened platelet reactivity have been reported in patients with COVID-19. Most of the micro-thrombi demonstrated in lungs were of in situ thrombosis in nature. Thus, in the presence of diffuse alveolar damage (endotheliitis), localized very high inflammation, and hypercoagulability seem to play the dominant role in the generation of thrombi with platelet activation facilitating the inflammation and coagulation activation processes.

Thrombotic events in COVID-19 have been described as immunothrombosis where innate immunity responses triggered by viral pathogens and diffuse alveolar damage (endotheliitis) occurs to arrest the spread and survival of the invading virus. During this process, activated neutrophils release tissue factor and NETs, and degrade endogenous anticoagulants thereby facilitating inflammation, induce hypercoagulability and subsequently the generation of fibrin rich thrombi. NETs directly activate platelets that further increase the inflammation and activation of coagulation by expressing surface receptors such as p-selectin and enhancing the formation of procoagulant platelet-neutrophil aggregates and release of NETs in a vicious cycle. Thus, the direct role of platelets may be modest in COVID-19, unlike coronary thrombosis. Since COVID-19 can be a rapidly progressive disease with a duration of a few weeks in selected patients, the timing of initiation, strength and route of antiplatelet therapy is critical to achieve a desirable and measurable clinical benefit. The role of potent antiplatelet therapy has not been sufficiently studied in COVID-19 to rule out potential benefits.

## Antithrombotic therapy clinical trials

A summary of completed, terminated, active-recruiting, and active- not recruiting clinical trials of antithrombotic therapy in patients with COVID-19 can be found in Tables 3 and 4 [164, 170, 171, 191–193, 196–205].

**Table 3:** Antiplatelet Treatment Studies in Patients with COVID-19

	Type of study	Population	Results
Observational studies			
TARGET-COVID, Sub-analysis (185)	Observational study	120 hospitalized COVID-19 patients and patients without COVID-19 29% of patients were on aspirin (81–325 mg/day)	U11-dh TxB_2_ was lower in aspirin-treated patients (p = 0.003) Inadequate therapeutic aspirin response observed in 91% of patients on 81 mg and 50% of patients on ≥ 162 mg daily aspirin
CRUSH COVID registry (186)	Retrospective, observational cohort study	314 hospitalized COVID-19 patients received aspirin within 24 h of admission; 98 no aspirin	Aspirin use associated with less mechanical ventilation (35.7% versus 48.4%, P = 0.03) and ICU admission (38.8% versus 51.0%, P = 0.04), but no crude association with in-hospital mortality (26.5% versus 23.2%, P = 0.51)
Veteran’s health administration study (187)	Retrospective, propensity score matching study	Hospitalized patients COVID-19 13,114 on prior aspirin and 13,114 no aspirin	Preexisting aspirin prescription was associated significant decrease in overall mortality at 14-days (OR = 0.38) and at 30-days (OR = 0.38)
Meizlish et al. (188)	Retrospective, propensity score matching study	638 hospitalized COVID-19 patients treated with aspirin	In‐hospital aspirin associated with a significantly lower in‐hospital death (HR = 0.52)
Merzon et al. (189)	Retrospective population‐based cross‐sectional study	73 COVID-19 positive patients who were on aspirin and 1548 not on aspirin	Patients on aspirin had a lower rate of COVID-19 (OR, 0.71; p = 0.04), and a shorter clinical duration of COVID-19 (19.8 ± 7.8 vs. 21.9 ± 7.9 days, p = 0.045)
Chow JH et al. (190)	Retrospective, propensity score matching study	6,781 COVID-19 patients on prehospital antiplatelet therapy and 10,566 patients on non‐antiplatelet therapy	Prehospital antiplatelet therapy was associated with significantly lower in‐hospital mortality (HR = 0.81, p < 0.005)
National Institutes of Health’s National COVID Cohort Collaborative Data Enclave study (191)	Observational cohort study	13,795 patients were on early hospital aspirin use and 98,275 not on aspirin	Aspirin use was associated with lower 28-day in-hospital mortality (OR = 0.85; P < 0.001) and pulmonary embolism (OR = 0.71, p = 0.004), similar gastrointestinal hemorrhage, cerebral hemorrhage or blood transfusion
Meta-analysis of observational studies (192)	23 pooled observational studies	87,824 patients	Antiplatelet therapy was associated with lower risk of mortality (OR = 0.72)
Randomized clinical trials			
RECOVERY Trial (NCT04381936) (193)	Randomized clinical trial	7351 patients received 150 mg/day aspirin and 7541 patients to receive usual care alone	No difference in 28-day mortality Aspirin therapy was associated with slightly shorter duration of hospitalization (median 8 days, vs 9 days,) a higher discharged from hospital alive within 28 days (75% vs 74%; rate ratio 1·06, p = 0·0062), lower rate of thrombotic events (absolute reduction = 0.6%) and higher major bleeding (absolute increase = 0.6%)
ACTIV-4A trial (NCT04505774) (194)	Open label, Bayesian, adaptive randomized clinical trial	293 non-critically ill patients treated with therapeutic dose of heparin plus a P2Y_12_ inhibitor vs. 269 patients on therapeutic dose of heparin only (usual care) for 14 days or until hospital discharge	Similar median number of organ support-free days -21 days (adjusted OR = 0.83) and non-significantly higher major bleeding in P2Y_12_ inhibitor group (adjusted OR = 3.31, P = 0.15)
ACTIV-4B trial (NCT04498273) (195)	Adaptive, randomized, double-blind, placebo-controlled trial	164 symptomatic clinically stable outpatients treated with 81 mg aspirin, 165 patients with 2.5 mg twice daily apixaban, 164 patients with 5 mg twice apixaban twice daily and 164 with placebo	Aspirin or apixaban compared with placebo did not reduce the rate of a composite clinical outcome of all-cause mortality, symptomatic venous or arterial thromboembolism, myocardial infarction, stroke, or hospitalization for cardiovascular or pulmonary cause. The study was terminated after enrollment of 9% of participants due to lower than anticipated event rate
REMAP-CAP (NCT02735707) (196)	Ongoing adaptive platform trial testing multiple interventions	565 critically ill patients received open-label aspirin, 455 received a P2Y_12_ receptor blocker, or 529 received no antiplatelet therapy for a maximum of 14 days in addition to anticoagulation thromboprophylaxis	There was similar median organ support–free days (7 in both the antiplatelet and control groups), median organ support-free days (14 days) and slightly higher proportions of patients surviving to hospital discharge (median-adjusted OR = 1.27; 96% posterior probability of efficacy), but higher major bleeding with antiplatelet therapy vs. control group (adjusted OR = 2.97, 99.4% probability of harm)
Meta-analysis of observational studies (192)	4 RCTs	–	Conflicting results. No benefit of adding antiplatelet therapy to the standard care, regardless of the baseline illness severity and concomitant anticoagulation intensity

*COVID-19* Coronavirus disease, *U11-dh TxB*_*2*_ urinary 11-dehydro thromboxane B_2_, *OR* Odds ratio, *RCT* randomized control trial

**Table 4:** Antithrombotic clinical trials in COVID-19

Study title	Status	Condition	Interventions	Results summary
NCT04367831 Intermediate or Prophylactic Dose Anticoagulation for Venous or Arterial Thromboembolism in Severe COVID-19: A Cluster Based Randomized Selection Trial (IMPROVE)	✔	Venous thrombosis or arterial thrombosis in COVID-19	-Enoxaparin Prophylactic Dose -Enoxaparin Intermediate Dose -Heparin Infusion -Heparin SC	Between intermediate and prophylactic doses no differences in all-cause mortality, major bleeding, ICU length of stay and clinically relevant arterial or venous thrombotic events noted
NCT04354155 COVID-19 Anticoagulation in Children—Thromboprophylaxis (COVAC-TP) Trial	✔	Venous thrombosis in COVID-19	-Enoxaparin Prefilled Syringe	Thromboprophylaxis with twice-daily enoxaparin is safe and warrants further investigation to assess efficacy
NCT04409834 Prevention of Arteriovenous Thrombotic Events in Critically Ill COVID-19 Patients Trial (COVID-PACT)	✔	Venous thromboembolism Or arterial thrombosis in COVID-19	-Heparin IV -Heparin SC -Enoxaparin 1 mg/kg -Enoxaparin 40 mg/0.4 mL Injectable Solution -Clopidogrel	In critically ill patients, full dose anticoagulation, but not clopidogrel reduced thrombotic complications, with an increase in bleeding
NCT04498273 COVID-19 Positive Outpatient Thrombosis Prevention in Adults Aged 40–80	✖	COVID-19	-Apixaban 2.5 mg -Apixaban 5 mg -Aspirin -Placebo	Among symptomatic clinically stable outpatients with COVID-19, treatment with aspirin or apixaban compared with placebo did not reduce the rate of a composite clinical outcome. However, the study was terminated after enrollment of 9% of participants because of an event rate lower than anticipated
NCT04401293 Systemic Anticoagulation with Full Dose Low Molecular Weight Heparin (LMWH) Vs. Prophylactic or Intermediate Dose LMWH in High Risk COVID-19 Patients (HEP-COVID Trial)	✔	COVID-19	-Enoxaparin -Prophylactic/ Intermediate Dose Enoxaparin	Therapeutic-dose LMWH reduced major thromboembolism and death compared with institutional standard heparin thromboprophylaxis among inpatients with COVID-19 with very elevated D-dimer levels. The treatment effect was not seen in ICU patients
NCT04558125 Low-Dose Tenecteplase in COVID-19 Diagnosed with Pulmonary Embolism	✖	Pulmonary embolism in COVID-19	-TNKase -Enoxaparin -Placebo	The study was terminated early and never unblinded. With only 2 subjects, statistical significance could never be demonstrated
NCT04829552 Prophylactic Versus Therapeutic Dose Anticoagulation in COVID-19 Infection at the Time of Admission to Critical Care Units	✔	COVID-19	Low dose: -Enoxaparin 40 mg daily -Heparin SC twice or three times daily High dose: - Enoxaparin 1 mg/kg twice daily - Enoxaparin 1.5 mg/kg daily -Heparin infusion	Treatment with high dose anticoagulation at the time of ICU/SDU admission was associated with decreased adjusted mortality among critically ill adult patients with COVID-19
NCT04406389 Anticoagulation in Critically Ill Patients With COVID-19 (The IMPACT Trial) (IMPACT)	◯	COVID-19	-Enoxaparin -Unfractionated Heparin - Fondaparinux -Argatroban	Ongoing trial
NCT04360824 COVID-19 Associated Coagulopathy	✔	COVID-19	-Enoxaparin Prophylactic Dose -Enoxaparin Intermediate Dose	In hospitalized adults with severe COVID-19, standard prophylactic dose and intermediate dose enoxaparin did not differ significantly in preventing death or thrombosis at 30 days
NCT04512079 FREEDOM COVID-19 Anticoagulation Strategy (FREEDOM COVID)	✔	COVID-19	-Enoxaparin -Apixaban	Among non-critically ill patients hospitalized with COVID-19, therapeutic anticoagulation did not reduce a 30-day composite endpoint; however, it was associated with a reduction in secondary outcomes of all-cause mortality and intubation compared with prophylactic anticoagulation
NCT04377997 Safety and Efficacy of Therapeutic Anticoagulation on Clinical Outcomes in Hospitalized Patients With COVID-19	◯	COVID-19 Cardiovascular Diseases	-Enoxaparin	Ongoing trial
NCT04505774 Accelerating COVID-19 Therapeutic Interventions and Vaccines 4 ACUTE (ACTIV-4A)	◯	COVID-19	-Therapeutic Heparin -Prophylactic Heparin -P2Y12	In critically ill patients with Covid-19, an initial strategy of therapeutic-dose anticoagulation with heparin did not result in a greater probability of survival to hospital discharge or a greater number of days free of cardiovascular or respiratory organ support than did usual-care pharmacologic thromboprophylaxis In noncritically ill patients with Covid-19, an initial strategy of therapeutic-dose anticoagulation with heparin increased the probability of survival to hospital discharge with reduced use of cardiovascular or respiratory organ support as compared with usual-care thromboprophylaxis
NCT04505592 Tenecteplase in patients with COVID-19	✔	COVID-19 Respiratory Failure ARDS	-Tenecteplase -Placebo	No significant difference noted in all-cause mortality and respiratory failure. Non conclusive results due to small sample size
NCT04372589 Antithrombotic Therapy to Ameliorate Complications of COVID-19 (ATTACC)	✔	COVID-19 Pneumonia	-Heparin	Not available
NCT04650087 COVID-19 Thrombosis Prevention Trials: Post-hospital Thromboprophylaxis	✔	• Covid19	• Apixaban 2.5 MG • Placebo	• Incidence of death and thromboembolism was low in this cohort. Study results noted to be imprecise and inconclusive
NCT04508023 A Study of Rivaroxaban to Reduce the Risk of Major Venous and Arterial Thrombotic Events, Hospitalization and Death in Medically Ill Outpatients With Acute, Symptomatic Coronavirus Disease 2019 (COVID-19) Infection	✖	• COVID-19	• Rivaroxaban • Placebo • Standard of care	• Study was terminated early. Rivaroxaban in non-hospitalized patients did not reduce risk of composite end point of venous or arterial thrombotic events, hospitalization, and death
NCT05226793 Medication Use Evaluation for Enoxaparin in Hospitalized COVID-19 Patients	◯	• Venous thromboembolism	• Observational study	• No results
NCT02735707 Randomized, Embedded, Multifactorial Adaptive Platform Trial for Community- Acquired Pneumonia (REMAP-CAP)	◯	• COVID-19	• Prophylactic intensity anticoagulation • Therapeutic intensity anticoagulation (LMWH, UFH) • Low dose thromboprophylaxis • Intermediate dose thromboprophylaxis	• In noncritically ill patients, therapeutic dose anticoagulation with heparin increased probability of survival to hospital discharge with reduced use of cardiovascular or respiratory organ support as compared with usual care thromboprophylaxis • In critically patients therapeutic dose anticoagulation with heparin did not result in greater probability of survival to hospital discharge or greater number of days free of cardiovascular or respiratory organ suppose than usual care pharmacologic thromboprophylaxis

Table 1 List of all clinical trials conducted in the United States to prevent or treat COVID-19 related thromboembolism. Obtained from clinicaltrials.gov

✔—Completed; ✖—Terminated; **◯**—Active-recruiting; **◯**—Active-not recruiting

## Current recommendations for thromboprophylaxis

### Acute phase of illness

COVID-19 is strongly associated with a heightened risk for VTE [206]. This risk is even higher among acutely or critically ill patients with multi-organ dysfunction. Head-to-head trials comparing efficacy of different doses and types of anticoagulation are currently underway and hence risk assessment for thrombosis and bleeding should be individualized prior to initiating treatment. Despite lack of RCT data, numerous national and international organizations have published clinical guidelines and released consensus statements on their recommendations of anticoagulation use in COVID-19. Many of these recommendations are inherently based on low certainty evidence and have gone through numerous changes and updates with the evolution of the pandemic.

National Institute of Health (NIH) issued updated guidelines on July 2023 on use of anticoagulation and antiplatelet in different clinical scenarios (COVID-19 Treatment Guidelines Panel. https://www.covid19treatmentguidelines.nih.gov/). By analyzing evidence from several studies, the COVID-19 treatment guidelines panel has recommended that patients on chronic anticoagulation or antiplatelet therapy continue these medications unless another contraindication arises (AIII) [207, 208]. In case of a confirmed thromboembolism or there is a high suspicion, patients with COVID-19 are recommended to be treated with therapeutic intensity anticoagulation (AIII) [171]. Both the NIH and the American Society of Hematology (ASH) recommend using prophylactic-intensity anticoagulation in patients with COVID-19 related critical illness requiring intensive level care including those requiring hemodynamic support, ventilatory support, renal replacement therapy or high-flow oxygen [170, 172, 209, 209, 210]. By contrast, patients who are acutely ill but not critically ill, and do not otherwise meet the criteria for receiving therapeutic anticoagulation, it is recommended to use prophylactic intensity anticoagulation for patients without confirmed or suspected thromboembolism [199, 210, 211]. In the MICHELLE (Rivaroxaban versus no anticoagulation for post-discharge thromboprophylaxis after hospitalization for COVID-19), 320 patients hospitalized with COVID-19 at increased risk for venous thromboembolism were treated with rivaroxaban or no anticoagulation. The primary efficacy outcome of composite of symptomatic or fatal venous thromboembolism, asymptomatic venous thromboembolism on bilateral lower-limb venous ultrasound and CT pulmonary angiogram, symptomatic arterial thromboembolism, and cardiovascular death at day 35 occurred in 3% of patients treated with rivaroxaban and 9% of patients treated with anticoagulation (relative risk 0·33, 95% CI 0·12–0·90; p = 0·0293). No major bleeding occurred in either study group. Prophylactic anticoagulation for patients that are being discharged from the hospital is no longer recommended by any society based on the results of MICHELLE and ACTIV-4c trials [212, 213].

## Recommended surveillance strategies for thromboembolism

The recognition and treatment of thromboembolism in COVID-19 remains a significant challenge for acutely and critically ill patients. There are limited data on the utility of routine ultrasound or computed tomography scans to diagnose lower extremity or pulmonary thromboembolism respectively. Despite the high frequency of thromboembolic events in COVID-19, no published data demonstrate the clinical utility of performing screening surveillance for deep venous thrombosis or pulmonary embolism in this population and thus no clinical guidance is available to follow.

The CDC panel states there is insufficient evidence from the COVID-19 treatment Guidelines Panel to recommend for or against routine screening for VTE in patients with COVID-19 who do not have signs or symptoms of VTE, regardless of the status of their coagulation markers. However, if there is rapid clinical deterioration of pulmonary, cardiac, or neurological function or sudden loss of peripheral perfusion, then evaluating for thromboembolic disease is recommended (AIII). Similarly, CHEST COVID-19 guidelines suggest against routine ultrasound screening for the detection of asymptomatic DVT in critically ill patients and instead recommends clinicians to have a low threshold for performing screening tests when there is a reasonable degree of suspicion for a VTE [214]. When compared to the CDC and the CHEST guidelines, the ASH COVID-19 guidelines do not specifically address the role of screening ultrasound in patents with COVID-19.

## CAD surveillance and ASCVD risk score adjustment in COVID-19

Patients with underlying coronary artery disease (CAD) are at a greater risk for severe COVID-19 disease. Additionally, COVID-19 can confer significant cardiovascular morbidity and mortality in patients with or without prior CVD [215]. Proposed mechanisms include inflammatory and thrombotic cascade activation and direct viral injury to myocytes or the vascular endothelium. Either could lead to worsening of the baseline atherosclerotic and structural abnormalities by causing plaque progression and destabilization resulting in disruption in coronary blood flow. It is therefore reasonable to consider patients who recover from acute or critical COVID-19 disease to be at risk for subclinical or possibly clinical cardiovascular disease. Patients who are disproportionately affected and at higher risk for cardiac injury are the elderly and frail, patients with metabolic syndrome, and those with preexisting CAD. Benefits of screening for residual cardiac dysfunction or atherosclerotic diseases in these groups of patients is currently unknown but should be considered to prevent morbidity and mortality. We believe that there are sufficient data to recommend aggressive risk factor modification in patients with prior COVID-19.

Most clinical trials, including observational studies and randomized controlled trials (RCTs) have primarily investigated the effects of antiplatelet therapy in hospitalized patients with acute COVID-19. Randomized trials, including COVID-PACT, ACTIV 4a and RECOVER did not demonstrate improved outcomes with aspirin or P2Y12 inhibitors [193, 200]. The long-term benefit of platelet-directed therapy in patients with COVID-19 has not been established.

Similarly, the potential risk of ASCVD-associated events and the benefit of antithrombotic therapy among patients with post-COVID conditions, also referred to as long-COVID, have not been determined. The NIH RECOVER (Researching COVID to Enhance Recovery) initiative will likely shed light on these important questions (recovercovid.org; accessed December 8, 2023).

## Summary and future directions

COVID-19 is the most recent addition to a list of acquired prothrombotic disorders or thrombophilias that displays several unique and distinct features. First, thrombosis can affect small, medium, and large vessels in the venous and arterial circulatory systems. Second, immune-mediated inflammatory responses can cause endothelial cell and to a lesser but likely meaningful degree glycocalyx injury. Third, thrombosis of the vasa vasorum impairs vascular integrity and reparative capacity. Fourth, a systemic prothrombotic state can persist beyond the acute infection stage either because of autoantibodies directed at intrinsic fibrinolytic or thrombosis regulating proteins or reservoirs (tissues and circulating mononuclear cells) of spike- 1 protein (or other SARS-CoV-2 structural proteins) that maintain low-level immuno-inflammation, prevent vascular endothelial cell repair, or elicit leukocyte activation signals for vascular injury and thrombosis.

Priorities for the medical and scientific communities to prevent, optimally manage, and perform targeted surveillance of patients with COVID-19 include the following:

1. an understanding of the immune, vascular endothelial cell, and leukocyte-specific signatures of COVID-19-associated thrombosis in the hyperacute, acute, and subacute stages of infection
2. An understanding of the roles of prothrombotic autoantibodies in the early convalescent stage,
3. Determination of the role of spike protein reservoir-mediated or associated immune, vascular, and leukocyte-driven thrombosis potential in the chronic phase.

## Data Availability

Not applicable.
